# Competing endogenous RNAs in human astrocytes: crosstalk and interacting networks in response to lipotoxicity

**DOI:** 10.3389/fnins.2023.1195840

**Published:** 2023-11-13

**Authors:** Natalia Gil-Jaramillo, Andrés Felipe Aristizábal-Pachón, María Alejandra Luque Aleman, Valentina González Gómez, Hans Deyvy Escobar Hurtado, Laura Camila Girón Pinto, Juan Sebastian Jaime Camacho, Alexis Felipe Rojas-Cruz, Yeimy González-Giraldo, Andrés Pinzón, Janneth González

**Affiliations:** ^1^Departamento de Nutrición y Bioquímica, Facultad de Ciencias, Pontificia Universidad Javeriana, Bogotá, Colombia; ^2^Laboratorio de Bioinformática y Biología de Sistemas, Universidad Nacional de Colombia, Bogotá, Colombia

**Keywords:** ceRNA (lncRNA–miRNA–mRNA), Transcriptome (RNA-seq), lipotoxicity, neurodegeneration, astrocyte

## Abstract

Neurodegenerative diseases (NDs) are characterized by a progressive deterioration of neuronal function, leading to motor and cognitive damage in patients. Astrocytes are essential for maintaining brain homeostasis, and their functional impairment is increasingly recognized as central to the etiology of various NDs. Such impairment can be induced by toxic insults with palmitic acid (PA), a common fatty acid, that disrupts autophagy, increases reactive oxygen species, and triggers inflammation. Although the effects of PA on astrocytes have been addressed, most aspects of the dynamics of this fatty acid remain unknown. Additionally, there is still no model that satisfactorily explains how astroglia goes from being neuroprotective to neurotoxic. Current incomplete knowledge needs to be improved by the growing field of non-coding RNAs (ncRNAs), which is proven to be related to NDs, where the complexity of the interactions among these molecules and how they control other RNA expressions need to be addressed. In the present study, we present an extensive competing endogenous RNA (ceRNA) network using transcriptomic data from normal human astrocyte (NHA) cells exposed to PA lipotoxic conditions and experimentally validated data on ncRNA interaction. The obtained network contains 7 lncRNA transcripts, 38 miRNAs, and 239 mRNAs that showed enrichment in ND-related processes, such as fatty acid metabolism and biosynthesis, FoxO and TGF-β signaling pathways, prion diseases, apoptosis, and immune-related pathways. In addition, the transcriptomic profile was used to propose 22 potential key controllers lncRNA/miRNA/mRNA axes in ND mechanisms. The relevance of five of these axes was corroborated by the miRNA expression data obtained in other studies. MEG3 (ENST00000398461)/hsa-let-7d-5p/*ATF6B* axis showed importance in Parkinson’s and late Alzheimer’s diseases, while AC092687.3/hsa-let-7e-5p/[*SREBF2, FNIP1, PMAIP1*] and SDCBP2-AS1 (ENST00000446423)/hsa-miR-101-3p/*MAPK6* axes are probably related to Alzheimer’s disease development and pathology. The presented network and axes will help to understand the PA-induced mechanisms in astrocytes, leading to protection or injury in the CNS under lipotoxic conditions as part of the intricated cellular regulation influencing the pathology of different NDs. Furthermore, the five corroborated axes could be considered study targets for new pharmacologic treatments or as possible diagnostic molecules, contributing to improving the quality of life of millions worldwide.

## 1. Introduction

Neurodegenerative diseases (NDs) represent one of the most significant problems for human health since they are one of the leading causes of disability and premature death worldwide (Erkkinen et al., 2018; Gauthier et al., 2021). The major concerning NDs are Alzheimer’s disease (AD), Parkinson’s disease (PD), amyotrophic lateral sclerosis (ALS), and Huntington’s disease (HD), which carry the greatest economic and social burden worldwide (Przedborski et al., 2003; Sertbaş et al., 2014). NDs are characterized by the progressive and irreversible loss of neurons, affecting the normal functioning of the central nervous system (CNS; Dugger and Dickson, 2017). Patients with NDs present non-specific symptoms and high clinical variability in the early stages, making diagnostic specificity inefficient and imprecise, which does not allow for timely treatment (Erkkinen et al., 2018).

Different neurophysiological studies have shown the importance of alterations in the normal functions of astrocytes in the development of NDs (Bylicky et al., 2018; Siracusa et al., 2019; Lee et al., 2022). These glial cells are crucial for the proper functioning of the CNS, fulfilling essential functions in its energy metabolism (Ortiz-Rodriguez and Arevalo, 2020). One of the main astrocytic functions is actively contributing to the formation and maintenance of the blood–brain barrier (BBB), which separates peripheral blood circulation from the highly controlled CNS microenvironment (Abbott et al., 2006). Astrocytes also secrete neurotrophic factors to regulate synaptogenesis, neuronal differentiation, and survival (Ullian et al., 2004; Karki et al., 2014; Anderson et al., 2018; Bylicky et al., 2018). In addition, astrocytes maintain brain homeostasis during metabolic disturbances by sensing nutrients, hormones, and other metabolites (Rose et al., 2020). Therefore, astrocytes are a widely used model for the study of NDs, given their direct influence on brain function and their relationship with the development of this type of disease (Lee et al., 2022).

Current lifestyle with increased intake of hypercaloric foods and decreased physical activity is causing a dramatic augment in obesity rates observed worldwide (World Health Organization, 2021). Interestingly, obesity increases palmitate concentration in the cerebrospinal fluid (CSF) in humans and can induce memory impairment in mice (Melo et al., 2020). Furthermore, recent evidence has shown that the accumulation of saturated fatty acids in non-adipose tissues, a phenomenon known as lipotoxicity (Sorensen et al., 2010; Unger et al., 2010; Schaffer, 2016), can trigger a hypothalamic proinflammatory response (Cesar and Pisani, 2017). This state alters mitochondrial functionality, increasing the concentration of reactive oxygen species (ROS; Schönfeld et al., 2010; Schonfeld and Reiser, 2021). Moreover, this scenario can potentially decrease brain homeostasis and induce a potentially harmful astrocytic inflammatory response, leading to a decline in cognitive activities, the progress of dementia, and the development of diseases such as AD and PD (Gupta et al., 2012; Kempuraj et al., 2017; Ortiz-Rodriguez et al., 2019; Angarita-Rodríguez et al., 2022).

Palmitic acid (PA) is a saturated fatty acid that occurs naturally in the human body, constituting 20%–30% of fat stores, and is present in many commonly consumed foods (Carta et al., 2017). However, PA can become cytotoxic when the regulation of its metabolism is inadequate. After chronic exposure to high levels of fatty acids, a series of pathological responses are generated, increasing proinflammatory cytokine and ROS production, thus leading to oxidative stress (Schönfeld and Reiser, 2017). These processes give rise to neuroinflammation, in which microglial cells and astrocytes play a fundamental role (Nordengen et al., 2019; Soung and Klein, 2019). When the body’s antioxidant mechanisms do not control this situation, it promotes neuronal damage and the maintenance of inflammatory processes (Schonfeld and Reiser, 2021).

Although the exact mechanisms related to a lipotoxic event have not been fully characterized, molecular and functional changes have been evidenced in astrocytes, providing the basis for the study of the underlying mechanisms that could be present in the development and progression of NDs (Ortiz-Rodriguez et al., 2019; Martin-Jiménez et al., 2020). Additionally, despite different studies finding some of the factors that lead to astrocytes going from being neuroprotective to neurotoxic (Liddelow et al., 2017; Clarke et al., 2018; Yun et al., 2018; Guttenplan et al., 2020, 2021; Wang and Li, 2023), there is still the need to understand the molecular background and key points of these processes. Current incomplete knowledge ought to be improved by the growing field of non-coding RNAs (ncRNAs), which is proven to be related to NDs (García-Fonseca et al., 2021), whose role in their development and progression is not yet understood. The reason is that complex regulatory networks are related to this process, and current overly simplistic approaches do not have the capacity to recognize and understand them.

Competing endogenous RNA (ceRNA) networks represent the complex crosstalk of RNAs through their miRNA-biding sites. These ceRNA networks define the way RNA molecules (lncRNAs, miRNAs, and mRNAs) regulate each other, controlling the final gene expression pattern (Salmena et al., 2011). Approximately 20,000 protein-coding genes and 19,000 pseudogenes and the increasing number of lncRNA transcripts identified in the human genome are densely covered in miRNA-biding sites, demonstrating how intricated this regulation can be (Friedman et al., 2009). Interestingly, the involvement of ceRNA networks has been observed in NDs, including AD, PD, and ALS (Zhang X. et al., 2020; Liu X. et al., 2021; Li et al., 2022).

Although there is the proven influence of some lncRNAs, circRNA, and miRNA in astrocyte function and dysfunction (Yi et al., 2019; Liao et al., 2020; Wan and Yang, 2020; Balint et al., 2021; Chen M. et al., 2021; Chen Z. et al., 2021; Gao et al., 2021; García-Fonseca et al., 2021; Nwokwu et al., 2022; Ramírez et al., 2022), the complexity of these networks remains unexplored in human astrocytes. According to that, we propose here a ceRNA network construction method, trying to understand the complex RNA crosstalk presented in astrocytes during stressful conditions. In this study, we focused on the ceRNA networks where lncRNAs, long non-coding RNAs with more than 200 nucleotides, can act as sponges over the miRNAs, ~22 nucleotide RNA sequences acting as negative gene regulators (Statello et al., 2020). Thus, when a lncRNA is upregulated, its related mRNAs will also be upregulated due to the lack of miRNA-directed degradation. In contrast, when a lncRNA is downregulated, more miRNA molecules could negatively regulate the mRNA expression. Consequently, different computational approaches were used to identify and characterize differentially expressed (DE) lncRNAs and mRNAs obtained from the transcriptomic analysis of human astrocyte cultures exposed or not to lipotoxic conditions. These DE lncRNAs and mRNAs were used to construct a ceRNA network, identifying potentially useful regulation axes, which could be used as prognostic biomarkers for the early ND diagnosis and targets for implementing effective therapies.

## 2. Materials and methods

### 2.1. Differentially expressed lncRNAs and mRNAs

Normal human astrocytes (NHA, Lonza Cat # CC-2565) from three different batches (#0000612736, #00005656712, and #0000514417) were cultured in ABM medium (Lonza, Basel, Switzerland) and SingleQuots supplements (Lonza, Basel, Switzerland) for 12 days at 37°C, humidified atmosphere and 5% CO_2_, plating 5 × 10^3^ cells/cm^2^. PA treatment was conducted following the protocols previously described (Martin-Jiménez et al., 2020; Rojas-Cruz et al., 2023). Briefly, NHA cells were starved in serum-free DMEM (Lonza, Basel, Switzerland) for 6 h, washed with 1X PBS, and cultured for 24 h in serum-free DMEM containing 2 mM PA, 1.35% of BSA (Sigma), and 2 mM carnitine (Sigma). Vehicle samples were cultured in serum-free DMEM only with 1.35% of BSA and 2 mM carnitine. RNA extraction, sequencing, and analysis were performed as described in the study by Angarita-Rodríguez et al. (2022). Differential expression analysis between PA-treated and vehicle (VH) cells was performed using the edgeR package (v3.36) in R software, normalizing gene for RNA composition among libraries by a trimmed mean of M-values (TMM; Robinson et al., 2010; Lun et al., 2016; Rojas-Cruz et al., 2023). DE genes presented |log_2_ fold change| > 1 and adjusted value of *p* < 0.05, which were calculated using Benjamini–Hochberg’s procedure for multiple comparison adjustment (Benjamini and Hochberg, 1995; Rojas-Cruz et al., 2023).

### 2.2. LncRNA–miRNA–mRNA interaction network

For lncRNA–miRNA interactions, each lncRNA transcript ID was searched in LncBase v3 from DIANA TOOLS (Paraskevopoulou et al., 2013; Karagkouni et al., 2020), considering the following filters to obtain high-confidence interactions: methods (HITS-CLIP, PAR-CLIP, CLEAR-CLIP, miR-CLIP, and Luciferase Reporter Assay), validation type (Direct), and species (*Homo sapiens*). Next, miRNAs were considered for the related pathway analysis in DIANA-mirPath v.3 (Vlachos et al., 2015a), employing the TarBase v.7 database (Vlachos et al., 2015b) with high-throughput methods and positive direct validation parameters. The miRNA-interacting mRNAs reported in neurodegenerative-related pathways were then compared with the one on the Starbase v2.0 (Li et al., 2014), using the filters of strict stringency (≥3) for CLIP data and medium stringency (≥2) for degradome data. After that, the lncRNA–miRNA–mRNA networks were recreated using Cytoscape 3.9.1 (Shannon et al., 2003).

### 2.3. Functional enrichment analysis

Enrichment analyses were conducted using two different approaches: First, all mRNAs included in the interaction networks were analyzed using Panther v.17 (Mi et al., 2013; Thomas et al., 2022), Benjamini–Hochberg FDR corrected value of *p* < 0.05, and ND-related terms. Pathway enrichment was verified using the transcriptomic data and Pathview (Luo and Brouwer, 2013). Second, each group of miRNAs that interact with the DE lncRNA transcripts was submitted to mirPath v.3 (Vlachos et al., 2015a), obtaining the significantly enriched KEGG pathways and GO terms. Only those terms with FDR corrected value of *p* ≤ 0.01 and reported relation with ND development were considered.

### 2.4. LncRNA–miRNA–mRNA axis selection

The data from *in silico* networks were compared with the expression profile in the previously obtained transcriptome, finding the more probable lncRNA–miRNA–mRNA axes under the PA lipotoxic condition. Selected axes include (a) upregulated lncRNA transcripts, downregulated miRNAs, and upregulated mRNAs, or (b) downregulated lncRNA transcripts, upregulated miRNAs, and downregulated mRNAs. No other options were considered. While we acknowledge the existence of potentiation relationships between lncRNAs and miRNAs, as well as the possibility of lncRNAs acting as precursors for their interacting miRNAs (Statello et al., 2020), this initial exploration will focus on the specific aspect of lncRNAs acting as miRNA sponges. This way, we only assessed the competing endogenous RNA axes that were more probable to be activated under our study condition. Then, to obtain the more probable axes regulating ND processes, we filtrated them by verifying the expression levels of the lncRNAs, miRNAs, and mRNAs in BioGPS (Wu et al., 2009), CNS microRNA profiles (Pomper et al., 2020), and miTED (Kavakiotis et al., 2022) databases. Additionally, the possible importance of the proposed axes was validated using the Gene Expression Omnibus database of NCBI (Edgar et al., 2002; Barrett et al., 2013), including the following ND-related studies: GSE155700 (Sproviero et al., 2021), GSE46131 (Hébert et al., 2013), GSE46579 (Leidinger et al., 2013), and GSE48552 (Lau et al., 2013).

### 2.5. Axis validation

For lncRNA and mRNA expression validation in the astrocytic model under PA lipotoxicity, total RNA was extracted using an RNeasy Mini Kit (Qiagen), following the manufacturer’s protocol. Then, samples were quantified with NanoDrop 2000 (Thermo Fisher Scientific), and their quality was assessed using Agilent 2,100 Bioanalyzer (Agilent Technologies). Only samples with an RNA Integrity Number (RIN) ≥ 8 were considered. After that, reverse transcription was performed using the GoTaq™ 2-Step RT-qPCR System (Promega) on a BioRad CFX real-time PCR system (BioRad) and the CFX Maestro™ Software (BioRad), without modification in the manufacturer’s instructions. Normalization for the 2^−ΔΔCT^ method was performed using the glyceraldehyde-3-phosphate dehydrogenase (*GAPDH*) gene, and an unpaired t-test was used to compare treatments with a value of *p* of <0.05 as significant. Primers were designed using primer blast (Ye et al., 2012). For MEG3 (ENST00000398461), the designed amplicon covered exons 2 and 3 to ensure the exclusive amplification of this transcript. Primers are listed in Supplementary Table 1.

## 3. Results

### 3.1. The ceRNA network controlling the astrocytic response to lipotoxicity

NHA cells were used as the model to understand the implications of PA metabolic imbalance and the possible consequences in the CNS of the cellular mechanisms activated in an astrocytic lipotoxic response. Under high PA concentrations, these cells presented 1,008 DE genes and demonstrated enrichment in immune activation pathways (Rojas-Cruz et al., 2023; Supplementary Table 2). Interestingly, 17 DE lncRNA transcripts were obtained when comparing PA-treated and vehicle (VH) cells, some of them previously related to ND processes (Table 1).

**Table 1:** Differentially expressed lncRNA transcripts between palmitic acid (PA) vs. vehicle (VH) astrocytes.

Transcript ID	Symbol	Status	Log_2_ fold change	p_adj_*	Possible ND-related function
ENST00000334146	MIR22HG	Upregulated	2.304	3.00 × 10^−5^	Regulates FoxO and TGF-β signaling pathways (Xu et al., 2020; Chen et al., 2022)
ENST00000375633	SNHG32	Upregulated	1.331	3.25 × 10^−2^	Promotes autophagy-induced neuronal cell apoptosis (Cao et al., 2020)
ENST00000446423	SDCBP2-AS1	Upregulated	2.169	6.22 × 10^−6^	Possible protection against apoptosis (Liu et al., 2021)
ENST00000553954		Upregulated	1.568	2.23 × 10^−2^	Unknown
ENST00000606907	AC092687.3	Upregulated	2.518	1.26 × 10^−4^	Unknown
ENST00000612303	NEAT1	Upregulated	1.538	1.32 × 10^−4^	Astrocyte activation and inflammation response (Wan and Yang, 2020; Liu et al., 2021b)
ENST00000643276		Upregulated	1.212	8.64 × 10^−4^	Unknown
ENST00000648820	MEG3	Upregulated	4.440	3.33 × 10^−2^	Protects against apoptosis in an AD rat model (Yi et al., 2019). Furthermore, can induce neuronal death, autophagy, and functional impairment (Luo et al., 2020).
ENST00000688585		Upregulated	2.728	6.71 × 10^−6^	Unknown
ENST00000689147	SNHG1	Upregulated	1.461	8.75 × 10^−5^	Relieves microglia activation (He et al., 2021), but also can induce neuronal damage in PD (Wang et al., 2021a,c).
ENST00000444125	LINC01503	Downregulated	−1.298	7.42 × 10^−5^	Disrupts amyloidogenic and mTOR pathways (Garofalo et al., 2021).
ENST00000437764	SERTAD4-AS1	Downregulated	−1.739	1.08 × 10^−3^	Unknown
ENST00000660635		Downregulated	−1.059	2.47 × 10^−2^	Unknown
ENST00000522618	MEG3	Downregulated	−2.457	3.57 × 10^−2^	Protects against apoptosis in an AD rat model (Yi et al., 2019). Furthermore, can induce neuronal death, autophagy, and functional impairment (Luo et al., 2020).
ENST00000624461		Downregulated	−1.853	2.74 × 10^−2^	Unknown
ENST00000398461	MEG3	Downregulated	−1.032	1.30 × 10^−2^	Protects against apoptosis in an AD rat model (Yi et al., 2019). Furthermore, can induce neuronal death, autophagy, and functional impairment (Luo et al., 2020).
ENST00000610851	MALAT1	Downregulated	−1.031	2.51 × 10^−2^	Related to the pathogenesis of ALS (Liu et al., 2021a).

^*^FDR adjustment was conducted by the Benjamini–Hochberg procedure.

**Figure 1:**
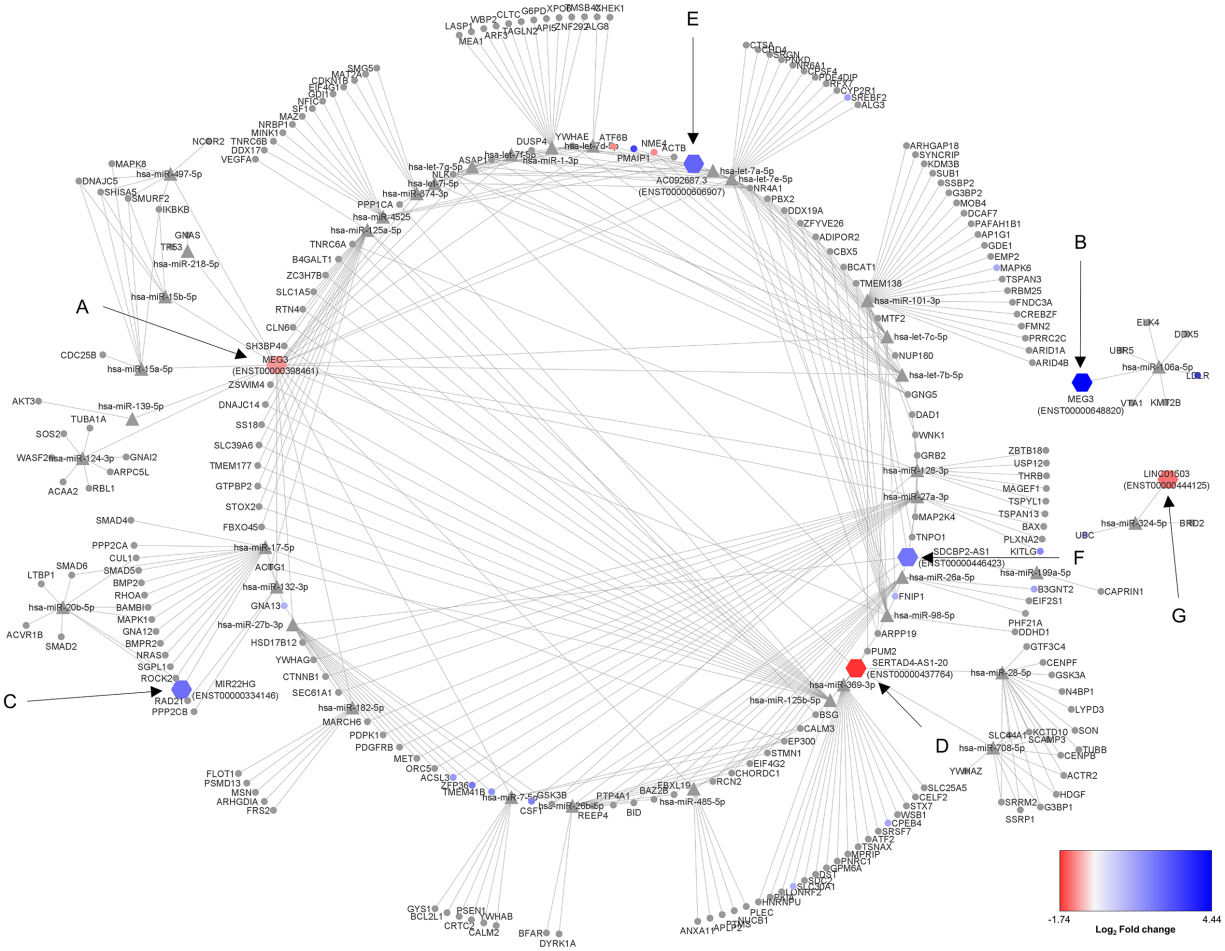
Regulatory ceRNA network activated in human astrocytes during a lipotoxic condition. The 17 differentially expressed (DE) lncRNA transcripts under a PA lipotoxic challenge were used to build an *in silico* predicted ceRNA network, applying CLIP data and degradome filters in Diana tools and Starbase databases. Only seven of them presented miRNA-interacting data reported in LncBase (showed as hexagon-shaped nodes): **(A)** MEG3 (ENST00000398461), **(B)** MEG3 (ENST00000648820), **(C)** MIR22HG (ENST00000334146), **(D)** SERTAD4-AS1-201 (ENST00000437764), **(E)** AC092687.3 (ENST0000606907), **(F)** SDCBP2-AS1 (ENST00000446423), **(G)** LINC01503 (ENST00000444125). The neurodegenerative disease (ND)-related miRNAs (triangle-shaped nodes) were considered for this network. The miRNA-interacting mRNAs are shown as circle-shaped nodes. Log_2_ fold change is displayed through node color, with dark blue for higher values, dark red for lower values, and gray for genes without differential expression data in the transcriptome.

From the 17 DE lncRNA obtained transcripts, only seven had reported interaction with miRNAs in LncBase. These transcripts were considered to obtain an *in silico* lncRNA/miRNA/mRNA network using the experimentally validated data from LncBase and Starbase. Additionally, transcriptomic data were also considered in the network to understand the resulting dynamics of the involved RNA molecules, demonstrating complex interactions among seven lncRNA transcripts, 38 miRNAs, and 239 mRNAs (Figure 1; Table 2).

**Table 2:** List of lncRNA–miRNA–mRNA interactions in human astrocytes under PA lipotoxic conditions.

lncRNA	miRNAs	mRNAs	lncRNA	miRNAs	mRNAs
MEG3 (ENST00000398461)	hsa-let-7a-5p	*ASAP1*	SERTAD4-AS1-20 (ENST00000437764)	hsa-miR-125a-5p	*ACTG1*
		*NLK*			*B4GALT1*
		*NR4A1*			*BSG*
		*YWHAE*			*CLN6*
	hsa-let-7b-5p	*ADIPOR2*			*DNAJC14*
		*ASAP1*			*FBXO45*
		***ATF6B***			*GTPBP2*
		*DUSP4*			*RTN4*
		*NLK*			*SH3BP4*
		***NME4***			*SLC1A5*
		*NR4A1*			*SLC39A6*
		*NUP160*			*SS18*
		***PMAIP1***			*STOX2*
		*YWHAE*			*TMEM177*
	hsa-let-7c-5p	*ADIPOR2*			*TNRC6A*
		*ARPP19*			*VEGFA*
		*BCAT1*			*ZC3H7B*
		*CBX5*			*ZSWIM4*
		*DDX19A*		hsa-miR-125b-5p	*ACTG1*
		***FNIP1***			*B4GALT1*
		*MTF2*			*BSG*
		*NLK*			*CLN6*
		*NR4A1*			*DNAJC14*
		*PBX2*			*FBXO45*
		*TMEM138*			*GTPBP2*
		*ZFYVE26*			*HNRNPU*
	hsa-let-7d-5p	*ALG8*			*PPP1CA*
		*ASAP1*			*RTN4*
		***ATF6B***			*SH3BP4*
		*CHEK1*			*SLC1A5*
		*NLK*			*SLC39A6*
		*YWHAE*			*SS18*
	hsa-let-7e-5p	*ADIPOR2*			*STOX2*
		*ALG3*			*TMEM177*
		*ARPP19*			*TNRC6A*
		*ASAP1*			*ZC3H7B*
		***ATF6B***			*ZSWIM4*
		*CBX5*		hsa-miR-28-5p	*ACTR2*
		*CHD4*			*CENPB*
		*CPSF4*			*CENPF*
		*CTSA*			*G3BP1*
		*CYP2R1*			*GSK3A*
		*DDX19A*			*GTF3C4*
		*DUSP4*			*HDGF*
		***FNIP1***			*KCTD10*
		*GNG5*			*LYPD3*
		*MTF2*			*N4BP1*
		*NLK*			*SCAMP3*
		*NR4A1*			*SLC44A1*
		*NR6A1*			*SON*
		*NUP160*			*SRRM2*
		*PDE4DIP*			*SSRP1*
		***PMAIP1***			*TUBB*
		*PNKD*		hsa-miR-369-3p	*ARPP19*
		*RFX7*			*ATF2*
		***SREBF2***			*CELF2*
		*SRGN*			***CPEB4***
		*TMEM138*			*DST*
		*YWHAE*			*GPM6A*
		*ZFYVE26*			*LONRF2*
	hsa-let-7f-5p	*ASAP1*			*MPRIP*
		*DUSP4*			*PKIA*
		*GNG5*			*PNRC1*
		*NLK*			*PUM2*
		*NR4A1*			*SDC2*
		*YWHAE*			*SLC25A5*
	hsa-let-7 g-5p	*ASAP1*			***SLC30A1***
		***ATF6B***			*SRSF7*
		*DUSP4*			*STX7*
		*GNG5*			*TSNAX*
		*NLK*			*WSB1*
		*NR4A1*		hsa-miR-708-5p	*ACTR2*
		*YWHAE*			*CENPB*
	hsa-let-7i-5p	*ASAP1*			*G3BP1*
		*DUSP4*			*HDGF*
		*GNG5*			*KCTD10*
		*NLK*			*SCAMP3*
		*NR4A1*			*SLC44A1*
		*YWHAE*			*SRRM2*
	hsa-miR-124-3p	*ACAA2*			*SSRP1*
		*ARPC5L*			*YWHAZ*
		*GNAI2*	AC092687.3 (ENST00000606907)	hsa-let-7e-5p	*ADIPOR2*
		*RBL1*			*ALG3*
		*SOS2*			*ARPP19*
		*TUBA1A*			*ASAP1*
		*WASF2*			***ATF6B***
	hsa-miR-128-3p	*DAD1*			*CBX5*
		*GRB2*			*CHD4*
		*GSK3B*			*CPSF4*
		*MAGEF1*			*CTSA*
		*MAP2K4*			*CYP2R1*
		*THRB*			*DDX19A*
		*TNPO1*			*DUSP4*
		*TNRC6A*			***FNIP1***
		*TSPAN13*			*GNG5*
		*TSPYL1*			*MTF2*
		*USP12*			*NLK*
		*WNK1*			*NR4A1*
		*ZBTB18*			*NR6A1*
	hsa-miR-132-3p	*ACTG1*			*NUP160*
		*PPP2CB*			*PDE4DIP*
		*RAD21*			***PMAIP1***
	hsa-miR-15a-5p	*CDC25B*			*PNKD*
		*DNAJC5*			*RFX7*
		*IKBKB*			***SREBF2***
		*MAPK8*			*SRGN*
		*SHISA5*			*TMEM138*
		*SMURF2*			*YWHAE*
	hsa-miR-15b-5p	*DNAJC5*			*ZFYVE26*
		*IKBKB*		hsa-miR-1-3p	*ACTB*
		*SHISA5*			*API5*
		*SMURF2*			*ARF3*
	hsa-miR-182-5p	*ARHGDIA*			*CLTC*
		*FLOT1*			*G6PD*
		*FRS2*			*LASP1*
		***GNA13***			*MEA1*
		*MSN*			*TAGLN2*
		*PSMD13*			*TMSB4X*
	hsa-miR-27a-3p	***ACSL3***			*WBP2*
		*BAX*			*XPO6*
		***CSF1***			*ZNF292*
		*CTNNB1*		hsa-miR-4525	*ACTB*
		*GRB2*			*DDX17*
		*HSD17B12*			*GDI1*
		***KITLG***			*MAZ*
		*MAP2K4*			*MINK1*
		*MARCH6*			*NFIC*
		*MET*			***NME4***
		*ORC5*			*NRBP1*
		*PDGFRB*			*SF1*
		*PDPK1*			*TNRC6B*
		*PLXNA2*	SDCBP2-AS1 (ENST00000446423)	hsa-miR-101-3p	*AP1G1*
		*SEC61A1*			*ARHGAP18*
		***TMEM41B***			*ARID1A*
		*YWHAG*			*ARID4B*
		***ZFP36***			*CREBZF*
	hsa-miR-27b-3p	***ACSL3***			*DAD1*
		***CSF1***			*DCAF7*
		*CTNNB1*			*EMP2*
		*GSK3B*			*FMN2*
		*HSD17B12*			*FNDC3A*
		*MARCH6*			*G3BP2*
		*MET*			*GDE1*
		*ORC5*			*KDM3B*
		*PDGFRB*			***MAPK6***
		*PDPK1*			*MOB4*
		*SEC61A1*			*PAFAH1B1*
		***TMEM41B***			*PRRC2C*
		*YWHAG*			*PUM2*
		***ZFP36***			*RBM25*
	hsa-miR-485-5p	*ANXA11*			*SSBP2*
		*APLP2*			*SUB1*
		*BSG*			*SYNCRIP*
		*CALM3*			*TNPO1*
		*NUCB1*			*TSPAN3*
		*PLEC*			*WNK1*
		*PTMS*		hsa-miR-199a-5p	*CAPRIN1*
	hsa-miR-497-5p	*DNAJC5*		hsa-miR-26a-5p	***B3GNT2***
		*IKBKB*			*BAZ2B*
		*MAPK8*			*BID*
		*NCOR2*			*CHORDC1*
		*SHISA5*			*EIF2S1*
		*SMURF2*			*EIF4G2*
	hsa-miR-7-5p	*BCL2L1*			*EP300*
		*CALM2*			*FBXL19*
		*CALM3*			***FNIP1***
		*CRTC2*			*GSK3B*
		*EP300*			*PHF21A*
		*GYS1*			*PTP4A1*
		*PSEN1*			*RCN2*
		*YWHAB*			*REEP4*
	hsa-miR-874-3p	*CDKN1B*			*STMN1*
		*EIF4G1*		hsa-miR-26b-5p	*BAZ2B*
		*MAT2A*			*BFAR*
		*PPP1CA*			*BID*
		*SMG5*			*CHORDC1*
	hsa-miR-98-5p	*ADIPOR2*			*DYRK1A*
		*ARPP19*			*EIF4G2*
		*BCAT1*			*EP300*
		*CBX5*			*FBXL19*
		*DDHD1*			*GSK3B*
		*DDX19A*			*PTP4A1*
		***FNIP1***			*RCN2*
		*MTF2*			*REEP4*
		*NLK*			*STMN1*
		*NR4A1*		hsa-miR-27a-3p	***ACSL3***
		*PBX2*			*BAX*
		*TMEM138*			***CSF1***
		*ZFYVE26*			*CTNNB1*
MEG3 (ENST00000648820)	hsa-miR-106a-5p	*DDX5*			*GRB2*
		*ELK4*			*HSD17B12*
		*KMT2B*			***KITLG***
		***LDLR***			*MAP2K4*
		*UBR5*			*MARCH6*
		*VTA1*			*MET*
MIR22HG (ENST00000334146)	hsa-miR-17-5p	*BAMBI*			*ORC5*
		*BMP2*			*PDGFRB*
		*BMPR2*			*PDPK1*
		*CUL1*			*PLXNA2*
		*EP300*			*SEC61A1*
		*GNA12*			***TMEM41B***
		***GNA13***			*YWHAG*
		*MAPK1*			***ZFP36***
		*NRAS*		hsa-miR-27b-3p	***ACSL3***
		*PPP2CA*			***CSF1***
		*RHOA*			*CTNNB1*
		*ROCK2*			*GSK3B*
		*SGPL1*			*HSD17B12*
		*SMAD4*			*MARCH6*
		*SMAD5*			*MET*
		*SMAD6*			*ORC5*
	hsa-miR-20b-5p	*ACVR1B*			*PDGFRB*
		*BAMBI*			*PDPK1*
		*CUL1*			*SEC61A1*
		*LTBP1*			***TMEM41B***
		*MAPK1*			*YWHAG*
		*ROCK2*			***ZFP36***
		*SMAD2*	LINC01503 (ENST00000444125)	hsa-miR-324-5p	*BRD2*
		*SMAD6*			***UBC***

RNA transcripts are displayed in modules, presenting the miRNAs interacting with each lncRNA and the mRNAs interacting with each miRNA. Upregulated genes in the transcriptome are shown in blue, while downregulated genes are shown in red.

First, transcript ENST00000398461 of the MEG3 lncRNA presented 23 miRNAs with reported interaction with mRNAs associated with ND according to mirPath (Figure 1A). Among the reported mRNAs, 11 of them showed differential regulation in astrocytes under the studied condition. *NME4* and *ATF6B* mRNAs were downregulated, as well as this MEG3 transcript. Interestingly, transcript ENST00000648820 of the MEG3 lncRNA was upregulated, presenting known interaction only with hsa-miR-106a-5p, and one upregulated mRNA: *LDLR* (Figure 1B).

In addition, the upregulated MIR22HG (ENST00000334146) showed interaction with hsa-miR-17-5p and hsa-miR-20b-5p, regulating a total of 19 genes, among which is *GNA13*, upregulated according to our transcriptome data (Figure 1C). Moreover, the downregulated SERTAD4-AS1-201 (ENST00000437764) had reported interaction with hsa-miR-125a-5p, hsa-miR-125b-5p, hsa-miR-708-5p, hsa-miR-369-3p, and hsa-miR-28-5p, interacting with 55 genes in total. Among these related genes, *CPEB4* and *SLC30A1* were upregulated in the transcriptome (Figure 1D). Additionally, the upregulated AC092687.3 (ENST0000606907) interacted with hsa-miR4525, hsa-miR-1-3p, and hsa-let-7e-5p, acting over 51 genes. Five of these potentially regulated genes were also found in the transcriptome (Figure 1E).

Furthermore, SDCBP2-AS1 (ENST00000446423) showed interaction with hsa-miR-199a-5p, hsa-miR-101-3p, hsa-miR-26a-5p, hsa-miR-26b-5p, hsa-miR-27a-3p, and hsa-miR-27b-3p. These miRNAs could regulate the degradation of 60 mRNAs, eight of them being DE, according to the transcriptome data (Figure 1F). Moreover, LINC01503 (transcript ENST00000444125) showed interaction with miR-324-5p, which has a reported regulation over the upregulated *UBC* mRNA (Figure 1G).

### 3.2. Inflammation, apoptosis, and cell development/differentiation as central pathways controlled by individual lncRNA transcripts

**Figure 2:**
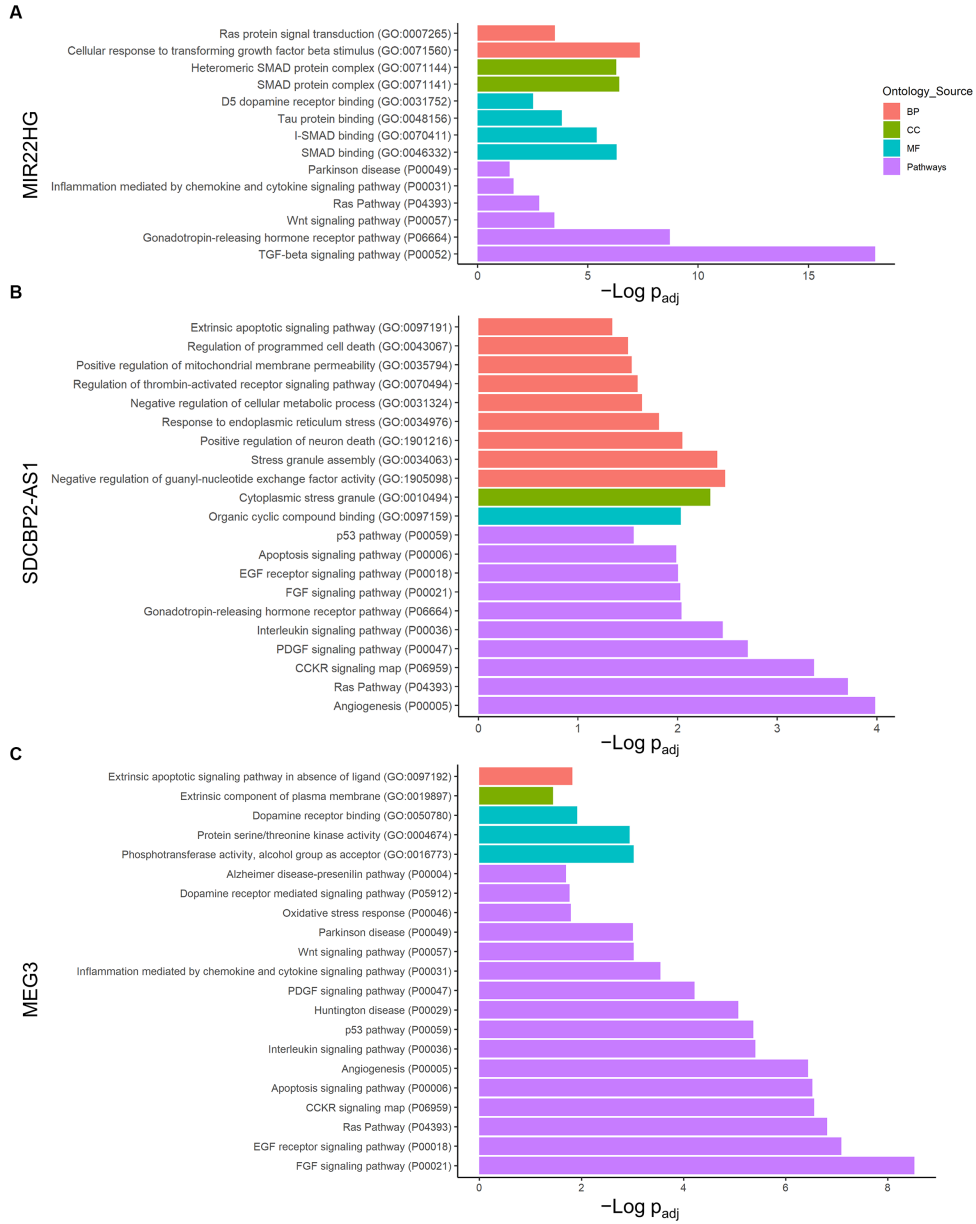
LncRNA transcript individual enrichment analysis. Genes in each lncRNA transcript network were analyzed using the Panther classification system. **(A)** MIR22HG (ENST00000334146), **(B)** SDCBP2-AS1 (ENST00000446423), and **(C)** MEG3 (ENST00000398461). Only terms with FDR-adjusted value of *p* < 0.05 and reported correlation with ND processes were considered. -Log (FDR-adjusted value of *p*) is displayed on the x-axis, and the ontology source of each term is shown according to bar color. BP, biological process; CC, cellular component; MF, molecular function.

Individual analysis of each lncRNA network allowed us to understand which pathways would be controlled by the DE transcripts found under lipotoxic conditions. Therefore, an enrichment analysis using Panther was conducted to identify the biological meaning of the potentially regulated genes in each lncRNA transcript network (Supplementary Table 3). In the case of MIR22HG (ENST00000334146), the TGF-β signaling and gonadotropin-releasing hormone receptor were the pathways with the lowest FDR-adjusted value of *p* (9.53 × 10^−19^ and 1.92 × 10^−9^, respectively). The GO terms significantly enriched by this lncRNA transcript are also related to SMAD-dependent TGF-β signaling (Figure 2A).

Furthermore, SDCBP2-AS1 (ENST00000446423) demonstrated a strong relationship with angiogenesis, cell proliferation and survival, and apoptosis pathways (Figure 2B). Angiogenesis, Ras pathway, and CCKR signaling map were the Panther pathways with the lowest FDR-adjusted value of *p* for this transcript (1.04 × 10^−4^, 1.96 × 10^−4^, and 4.25 × 10^−4^, respectively). Interestingly, the GO terms negative regulation of thrombin-activated receptor signaling pathway, negative regulation of guanyl nucleotide exchange factor activity, regulation of programmed cell death, stress granule assembly, heterocyclic compound binding, and cytoplasmic stress granule were enriched in this lncRNA.

In addition, in MEG3 (ENST00000398461), the fibroblast growth factor (FGF) signaling pathway showed the lowest FDR-adjusted value of *p* (3.02 × 10^−9^) and 8.66% of the term coverage (Figure 2C). Moreover, among the pathways in Panther, a strong relationship with inflammation, apoptosis, and specific NDs was found. Notably, dopamine receptor binding was the term with the highest coverage, where two of the four genes in the molecular function GO term were present in this MEG3 transcript (FDR-adjusted value of *p* = 1.02 × 10^−2^). The next term in coverage was extrinsic apoptotic signaling pathway in the absence of ligand, with 17.65% and FDR-adjusted value of *p* = 1.50 × 10^−2^.

On the other hand, transcripts MEG3 (ENST00000648820), LINC01503 (ENST00000444125), AC092687.3 (ENST00000606907), and SERTAD4-AS1 (ENST00000437764) did not show significantly overrepresented pathways in Panther.

### 3.3. Ras, angiogenesis, inflammation, and apoptosis are redundantly regulated pathways by both upregulated and downregulated lncRNA transcripts

**Figure 3:**
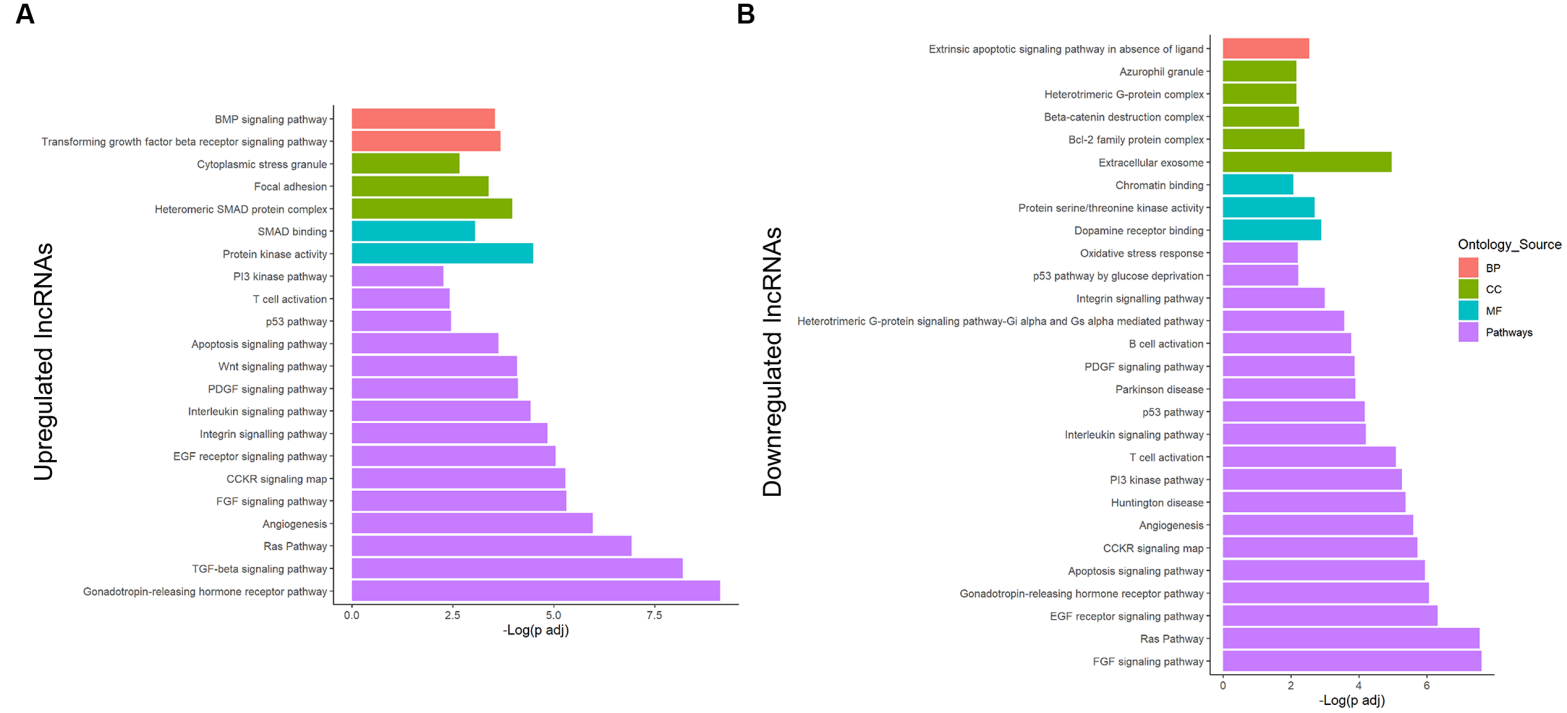
Enrichment analysis of the potentially regulated genes in the lncRNA–miRNA–mRNA network from astrocytes under a PA insult. Genes in the obtained network were analyzed using the Panther classification system for the upregulated **(A)** and downregulated **(B)** lncRNA transcripts. Only terms with FDR-adjusted value of *p* < 0.05 and reported correlation with ND processes were considered. −Log (FDR-adjusted value of *p*) is displayed on the x-axis, and the ontology source of each term is shown according to bar color. BP, biological process; CC, cellular component; MF, molecular function.

Additionally, Panther was also used for analyzing the whole network, assessing upregulated vs. downregulated transcripts (Figure 3; Supplementary Table 3), helping us to understand which processes would be activated or repressed under lipotoxicity. Altogether, the upregulated lncRNAs controlled pathways associated with gonadotropin-releasing hormone receptor I, inflammation, Ras, angiogenesis, apoptosis, and cell survival (Figure 3A). The SMAD-dependent TGF-β signaling pathway was highly enriched by the upregulated lncRNA transcripts. On the other hand, the FGF was the pathway with the lowest FDR-adjusted value of *p* in the downregulated lncRNAs (Figure 3B). Furthermore, the GO terms revealed interesting enrichments, including extracellular exosome, dopamine receptor binding, and azurophil granule. Ras pathway, angiogenesis, inflammation, and apoptosis were also relevant in the downregulated group.

### 3.4. Hippo and TGF-β signaling pathways are strongly controlled by the miRNAs in the ceRNA network

**Figure 4:**
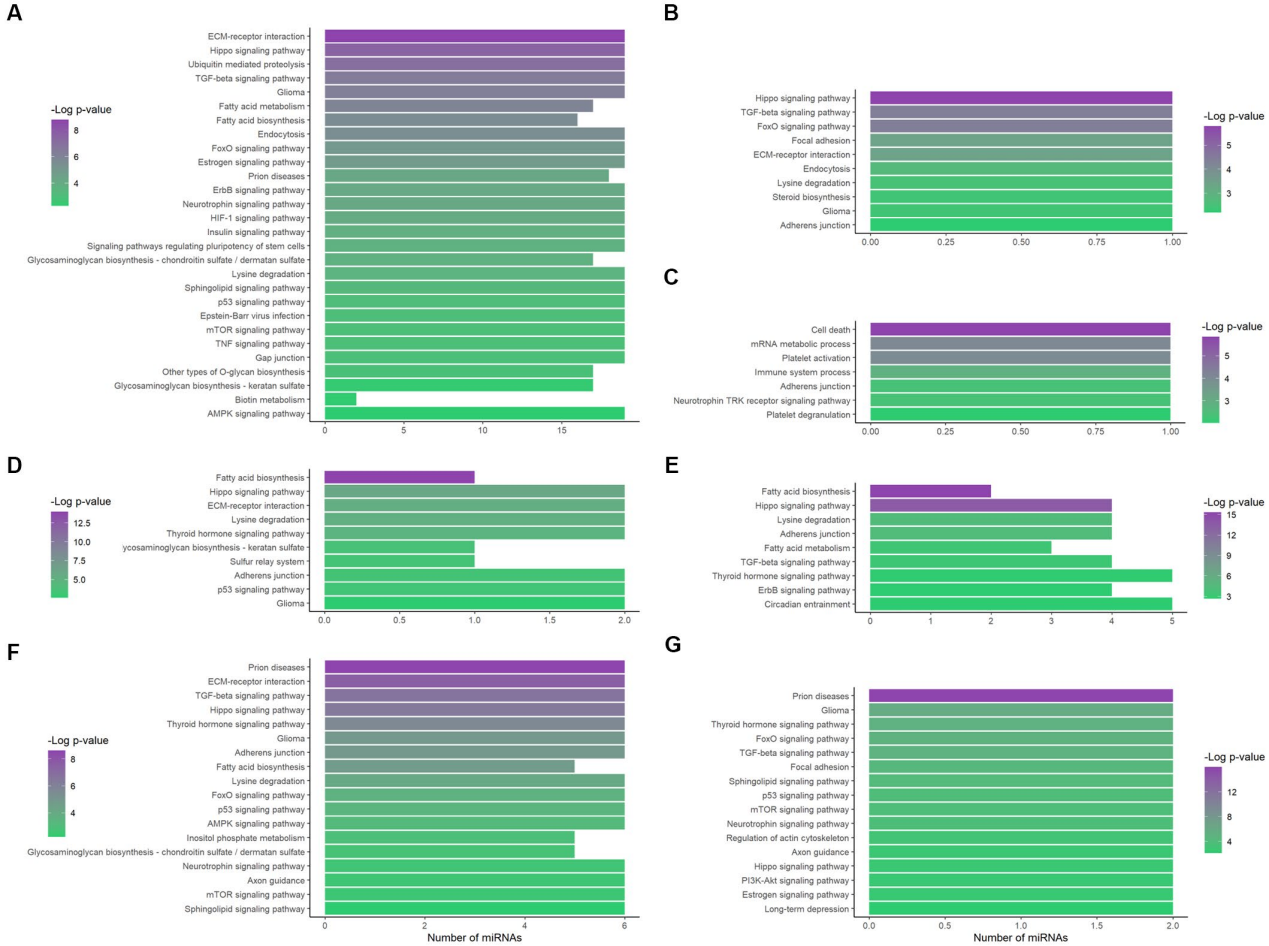
Functional enrichment analysis of the lncRNA-related miRNAs. The interacting miRNAs of MEG3 (ENST00000398461) **(A)**, MEG3 (ENST00000648820) **(B)**, LINC01503 (ENST00000444125) **(C)**, AC092687.3 (ENST00000606907) **(D)**, SERTAD4-AS1 (ENST00000437764) **(E)**, SDCBP2-AS1 (ENST00000446423) **(F)**, and MIR22HG (ENST00000334146) **(G)** were analyzed through mirPath, obtaining the overrepresented pathways. Only FDR-adjusted value of *p* ≤ 0.01, ND-related pathways are displayed here. The number of miRNAs that enrich each pathway is shown on the x-axis; − Log (p_adj_) is indicated by color according to the corresponding scale.

On the other hand, an alternative enrichment analysis was conducted by introducing the lncRNA-interacting miRNAs in the regulation network in the mirPath database. Individual enrichment analysis of the miRNAs related to MEG3 (ENST00000398461) showed that 26.4% of the enriched pathways are directly related to cancer (Supplementary Table 4). Furthermore, after filtrating the ND-associated pathways, our analysis revealed an important regulation in the extracellular matrix (ECM), with FDR-adjusted value of *p* = 1.65 × 10^−9^ and all the miRNAs involved (Figure 4A). Additionally, apoptosis, inflammation, and metabolism-related pathways, including fatty acids, were also overrepresented.

Additionally, the unique MEG3 (ENST00000648820)-associated miRNA, hsa-miR-106a-5p, presented 38 enriched pathways and 39.5% of them are related to cancer (Supplementary Table 4). In this case, Hippo and TGF-β signaling were also crucial pathways, with FDR-adjusted value of *p* = 1.64 × 10^−6^ and 4.03 × 10^−5^, respectively (Figure 4B).

Moreover, LINC01503 (ENST00000444125) showed interaction with hsa-miR-324-5p, which only had a reported enrichment for the adherens junction KEGG pathway, with p_adj_ = 4.47 × 10^−3^ and five related genes. However, 39 GO terms showed overrepresentation (Supplementary Table 4), some of them related to apoptosis, platelet activation, and the immune system (Figure 4C).

In addition, the three AC092687.3 (ENST00000606907)-interacting miRNAs demonstrated overrepresentation in 19 pathways, 26% of them with direct correlation to cancer (Supplementary Table 4). These miRNAs significantly enriched the fatty acid biosynthesis and Hippo signaling pathways, showing an FDR-adjusted value of *p* of 1.08 × 10^−14^ and 1.03 × 10^−6^, respectively (Figure 4D). Similarly, in the case of the five SERTAD4-AS1 (ENST00000437764)-related miRNAs, fatty acid biosynthesis presented the lowest FDR-adjusted value of *p* (4.85 × 10^−16^), followed by the Hippo signaling pathway (2.29 × 10^−14^; Figure 4E). These five miRNAs interacting with SERTAD4-AS1 reflected 15 enriched pathways, and 27% of them are directly related to cancer (Supplementary Table 4). Metabolism-related pathways were enriched in both lncRNA transcripts.

Six miRNAs demonstrated interaction with SDCBP2-AS1 (ENST00000446423). These miRNAs enriched 53 pathways according to mirPath, 18 of them directly related to cancer (Supplementary Table 4). Interestingly, prion diseases showed an important overrepresentation, with an FDR-adjusted value of *p* of 2.41 × 10^−9^. In addition, ECM–receptor interaction, TGF-β, and Hippo signaling, as well as metabolism pathways, were also overrepresented by these miRNAs (Figure 4F).

In the case of hsa-miR-17-5p and hsa-miR-20b-5p, which interacted with MIR22HG (ENST00000334146), 39.5% of the 38 enriched pathways were related to cancer (Supplementary Table 4). In this study, prion diseases were again highly overrepresented (FDR-adjusted value of *p* of 1.04 × 10^−16^), along with TGF-β and Hippo signaling pathways. Noteworthy, estrogen and long-term depression were enriched by these miRNAs (Figure 4G).

### 3.5. LncRNA–miRNA–mRNA axis expression in the CNS correlates with a biological role of controlling the astrocytic lipotoxic response

**Figure 5:**
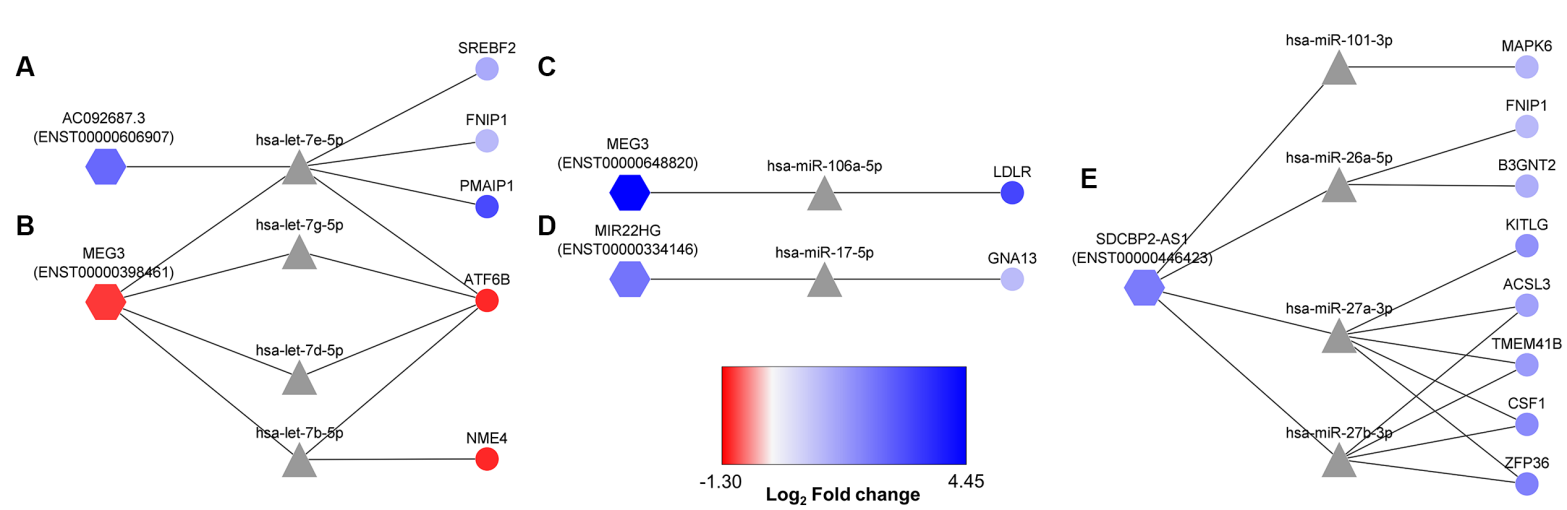
More probable lncRNA–miRNA–mRNA axes activated in human astrocytes under PA lipotoxic conditions. Selected axes contain lncRNAs (hexagon-shaped) and mRNAs (circle-shaped), both downregulated or both upregulated, as miRNAs (triangle-shaped) would direct target mRNA degradation. **(A)** AC092687.3 (ENST0000606907) related axes; **(B)** MEG3 (ENST00000398461) related axes; **(C)** MEG3 (ENST00000648820) related axis; **(D)** MIR22HG (ENST00000334146) related axis; **(E)** SDCBP2-AS1 (ENST00000446423) related axes. Log_2_ fold change is displayed through node color, with dark blue for higher values, dark red for lower values, and gray for genes without differential expression data in the transcriptome.

According to the *in silico*-obtained ceRNA network and considering all the possible lncRNA–miRNA–mRNA combinations, 22 axes were found as potential regulators when astrocytes are cultured in a high PA concentration (Figure 5). MEG3 (ENST00000398461) transcript was found among these potential axes. This lncRNA formed axes with hsa-let-7b-5p, hsa-let-7d-5p, hsa-let-7e-5p, and hsa-let-7 g-5p, regulating *NME4* and *ATF6B* gene expression. In addition, AC092687.3 (ENST00000606907) axes also involved the hsa-let-7e-5p miRNA, regulating the *SREBF2*, *FNIP1*, and *PMAIP1* expression (Figures 5A,B). However, AC092687.3 was upregulated under lipotoxic conditions, whereas MEG3 (ENST00000398461) was downregulated.

Furthermore, the upregulated ENST00000648820 transcript of MEG3 constituted an axis with hsa-miR-106a-5p, regulating *LDLR* expression (Figure 5C). Moreover, MIR22HG (ENST00000334146), which was upregulated in the transcriptome, interacted with hsa-miR17-5p, probably repressing the degradation of *GNA13* mRNA (Figure 5D). Finally, the upregulated SDCBP2-AS1 (ENST00000446423) formed 12 possible axes, regulating the degradation activity over *ACSL3*, *MAPK6*, *FNIP1*, *B3GNT2*, *TMEM41B*, *KITLG*, *CSF1*, and *ZFP36* (Figure 5E).

Additionally, we can observe the behavior of these RNA molecules relative to the median of total tissues in physiological conditions using BioGPS and CNS microRNA profiles, and even verify if they are related to any ND through the miTED database (Supplementary Table 5). This way, it is possible to corroborate if the RNAs in the axes are regularly expressed and can be differentially regulated in astrocytes and CNS. BioGPS showed MEG3 was a lncRNA highly expressed in the brain, while *NME4*, *ATF6B*, and *LDLR* showed a median expression. Regarding the miRNAs in CNS microRNA profiles, hsa-let-7b-5p showed the highest expression in astrocytes among the let-7 family. Interestingly, miTED demonstrated the importance of the let-7 family in NDs, being hsa-let-7d-5p and hsa-let-7 g-5p increased in PD. This expression profile would agree with the MEG3 (ENST00000398461)/hsa-let-7d-5p, hsa-let-7 g-5p/*ATF6B* axes in our hypothesis. On the other hand, the AC092687.3 (ENST00000606907)/ hsa-let-7e-5p/[*SREBF2*, *FNIP1*, *PMAIP1*] axes would not be supported by the miTED data.

MIR22HG showed a median expression in the brain, while *GNA13* showed an expression slightly above the median. Noteworthy, hsa-miR-17-5p was highly expressed in astrocytes, but its expression did not significatively change in NDs, according to miTED, which is also the case of hsa-miR-106a-5p in the MEG3 (ENST00000648820)/hsa-miR-106a-5p/*LDLR* axis. Therefore, miTED did not support the MEG3 (ENST00000648820)/hsa-miR-106a-5p/*LDLR* and MIR22HG (ENST00000334146)/hsa-miR-17-5p/*GNA13* axes.

In addition, SDCBP2-AS1 presented an expression more than 10 times the median in neurons and more than 3 times the median in astrocytes. Furthermore, miTED data agreed with the proposed SDCBP2-AS1 (ENST00000446423)/hsa-miR-27a-3p, hsa-miR-27b-3p axes, since hsa-miR-27a-3p and hsa-miR-27b-3p were reduced in hippocampal sclerosis ILAE type 1 and PD, respectively. Regarding the mRNAs involved in these axes, *KITLG*, *TMEM41B*, and *CSF1* had a median expression according to BioGPS. Moreover, *ACSL3* had an expression more than 3X the median in the prefrontal cortex, amygdala, hypothalamus, thalamus, and occipital lobe, and *ZFP36* presented a reduced expression in the CNS, except for the spinal cord, which is above the median expression.

Therefore, the data observed in the databases, with a basal expression of the lncRNAs and mRNAs of the axes, reinforce our transcriptomic data supporting the ceRNA network obtained here. In addition, these data demonstrate the fundamental role of these lncRNA, miRNA, and mRNA in astrocytes and CNS in general. Consequently, the dysregulation of these molecules due to stressful conditions, such as lipotoxicity, could be potentially harmful.

### 3.6. LncRNA–miRNA–mRNA axes may have a role in neurodegenerative processes

To further explore the importance of the miRNAs in the selected axes, we obtained the miRNA expression data from different studies related to NDs in the GEO database. Table 3 shows the miRNA differential expression profile in analyses of AD stages I and VI (GSE48552) and multiple NDs, including AD, PD, frontotemporal dementia (FTD), dementia with Lewy bodies (DLBs), hippocampal sclerosis of aging (HS), and sporadic ALS (GSE46131, GSE46579, and GSE155700).

**Table 3:** Expression profile of the selected miRNAs in ND-related studies obtained through the GEO database.

miRNA	GSE46131	GSE46579	GSE48552	GSE155700
hsa-let-7b-5p	Non-significant expression changes in any of the comparisons	Non-significant expression changes	Non-significant expression changes	Non-significant expression changes in any of the comparisons
hsa-let-7d-5p	Log_2_FC = 0.40, p_adj_ = 0.02 in AD VI vs. CTR	Log_2_FC = −0.42, p_adj_ = 0.02 in AD vs. CTR	Log_2_FC = −0.81, p_adj_ = 3.02 × 10^−5^ in AD VI vs. CTR	Non-significant expression changes in any of the comparisons
hsa-let-7e-5p	Non-significant expression changes in any of the comparisons	Log_2_FC = −0.72, p_adj_ = 1.55 × 10^−5^ in AD vs. CTR	Log_2_FC = −0.39, p_adj_ = 6.00 × 10^−4^ in AD VI vs. CTR	Non-significant expression changes in any of the comparisons
hsa-let-7 g-5p	Non-significant expression changes in any of the comparisons	Log_2_FC = −1.07, p_adj_ = 1.60 × 10^−6^ in AD vs. CTR	Log_2_FC = −0.48, p_adj_ = 2.03 × 10^−6^ in AD VI vs. CTR	Non-significant expression changes in any of the comparisons
hsa-miR-106a-5p	Non-significant expression changes in any of the comparisons	Non-significant expression changes	Non-significant expression changes	Non-significant expression changes in any of the comparisons
hsa-miR-17-5p	Log_2_FC = 0.60, p_adj_ = 0.03 in DLB vs. CTR and Log_2_FC = 0.92, p_adj_ = 6.85× 10^−3^ in HS aging vs. CTR	Non-significant expression changes	Log_2_FC = 0.98, p_adj_ = 1.08 × 10^−6^ in AD VI vs. CTR	Non-significant expression changes in any of the comparisons
hsa-miR-101-3p	Non-significant expression changes in any of the comparisons	Log_2_FC = −1.61, p_adj_ = 2.26 × 10^−5^ in AD vs. CTR	Log_2_FC = −0.37, p_adj_ = 3.14 × 10^−5^ in AD VI vs. CTR	Non-significant expression changes in any of the comparisons
hsa-miR-27a-3p	Non-significant expression changes in any of the comparisons	Non-significant expression changes	Non-significant expression changes	Non-significant expression changes in any of the comparisons
hsa-miR-27b-3p	Log_2_FC = 0.5, p_adj_ = 0.02 in DLB vs. CTR	Non-significant expression changes	Non-significant expression changes	Non-significant expression changes in any of the comparisons
hsa-miR-26a-5p	Non-significant expression changes in any of the comparisons	Non-significant expression changes	Log_2_FC = 0.80, p_adj_ = 6.67 × 10^−3^ in AD VI vs. CTR	Log_2_FC = −0.89, p_adj_ = 0.05 in PD vs. CTR

FC, Fold change; p_adj_, Benjamini–Hochberg FDR-adjusted value of p; AD, Alzheimer disease; DLB, dementia with Lewy bodies; HS aging, hippocampal sclerosis of aging; CTR, control.

According to these results, the GSE46131 study would demonstrate a slight increment in the expression of hsa-let-7d-5p in a late AD stage compared with control individuals. Therefore, data from the GSE46131 analysis would support our MEG3(ENST00000398461)/hsa-let-7d-5p/*ATF6B* axis hypothesis. However, the other ND conditions did not coincide with this hypothesis, showing negative regulation over the let-7 family. On the contrary, GSE46579 and GSE48552 studies would support the AC092687.3 (ENST00000606907)/hsa-let-7e-5p/[*PMAIP1*, *SREBF2*, *FNIP1*] axes.

Additionally, GSE46131 showed significative increments in the expression of hsa-miR-17-5p in individuals with DLB and HS compared with non-demented control subjects. In addition, GSE48552 revealed a rise of hsa-miR-17-5p expression in the early stages of AD. Both studies showed a medium increment in this miRNA expression, which is inconsistent with the expected behavior in axis MIR22HG (ENST00000334146)/miR-17-5p/*GNA13*.

Regarding the SDCBP2-AS1-related axes, hsa-miR-101-3p was consistently less expressed in AD according to GSE46579 and GSE48552, supporting the SDCBP2-AS1 (ENST00000446423)/hsa-miR-101-3p/*MAPK6* axis. Additionally, hsa-miR-27b-3p was increased in DLB (GSE46131 study), in disagreement with SDCBP2-AS1 (ENST00000446423)/hsa-miR-27b-3p/[*ACSL3*, *TMEM41B*, *CSF1*, *ZFP36*] axes. Moreover, only the GSE155700 study observed a negative regulation in hsa-miR-26a-5p in PD, which reinforces the SDCBP2-AS1 (ENST00000446423)/hsa-miR-26a-5p/[*FNIP1* and *B3GNT2*] hypothesis.

Thus, the MEG3 (ENST00000398461)/hsa-let-7d-5p/*ATF6B*, AC092687.3 (ENST0000606907)/hsa-let-7e-5p/*SREBF2*, AC092687.3 (ENST0000606907)/hsa-let-7e-5p/*FNIP1*, AC092687.3 (ENST0000606907)/hsa-let-7e-5p/*PMAIP1*, and SDCBP2-AS1 (ENST00000446423)/ hsa-miR-101-3p/*MAPK6* axes would be key in controlling processes related to AD and PD, according to the GSE48552, GSE46131, GSE46579, and GSE155700 studies.

### 3.7. Validation

**Figure 6:**
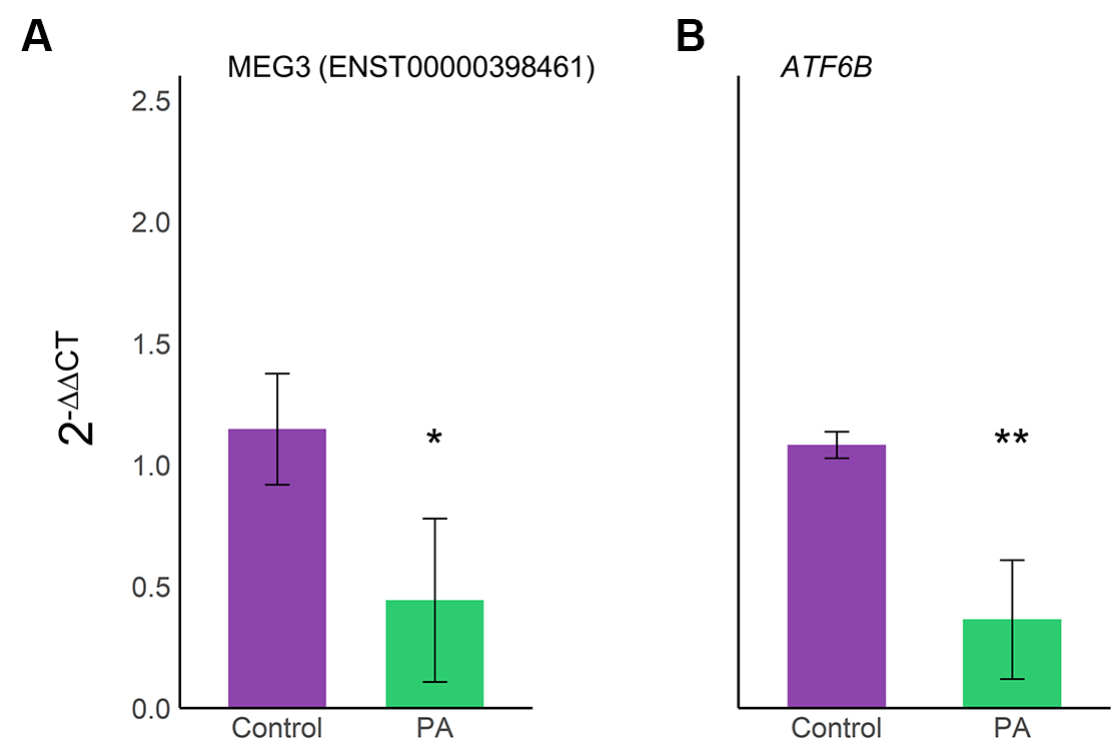
Validation of the lncRNA–miRNA–mRNA axis expression through RT-qPCR. Expression levels of the selected axis MEG3(ENST00000398461)/hsa-let-7d-5p/ATF6B were confirmed using RT-qPCR on three biological samples in triplicate. Bar plots display an average 2^−ΔΔCT^ ± SD of MEG3(ENST00000398461) **(A)** and *ATF6B* **(B)**. Violet, vehicle samples; green, PA-treated human astrocytes; ^*^p < 0.05; ^**^p < 0.01.

To validate the expression of selected transcripts on the proposed axes, MEG3(ENST00000398461) and *ATF6B* were quantified using RT-qPCR (Figure 6). Interestingly, MEG3 (ENST00000398461) expression correlated with our previous transcriptomic results, demonstrating that this specific transcript is downregulated in astrocytes under lipotoxic conditions (Figure 6A). Furthermore, *ATF6B* was also downregulated, as seen in the transcriptome (Figure 6B).

## 4. Discussion

The growing field of ncRNAs is demonstrating a fundamental role in neuroinflammation (Chen Z. et al., 2021), neurodegeneration (García-Fonseca et al., 2021), and microglia and astrocyte dysfunction (Chen M. et al., 2021). However, simplistic approaches do not have the capacity to understand the intricated regulation mechanisms among these ncRNAs. Computational methods are crucial for integrating data as complex as the ncRNA interaction networks, whose crosstalk defines gene expression under determinate conditions (Marques and Gama-Carvalho, 2022). In this study, we obtained the ceRNA network and 22 lncRNA/miRNA/mRNA axes, which potentially control the response of astrocytes against high PA concentrations by mixing transcriptomic data and an *in silico* approach. Interestingly, five of these axes were supported by the miRNA expression in databases with ND information and ND studies: MEG3 (ENST00000398461)/hsa-let-7d-5p/*ATF6B*, AC092687.3 (ENST0000606907)/hsa-let-7e-5p/*SREBF2*, AC092687.3 (ENST0000606907)/hsa-let-7e-5p/*FNIP1*, AC092687.3 (ENST0000606907)/hsa-let-7e-5p/*PMAIP1*, and SDCBP2-AS1 (ENST00000446423)/ hsa-miR-101-3p/*MAPK6*.

The present network, including 7 lncRNA transcripts, 38 miRNAs, and 239 mRNAs, would influence the astrocytic metabolism and inflammatory or stress processes. Astrocyte metabolism is strongly related to neurodegeneration due to the neuronal support given through nutrient transport, blood flow regulation, glycogen storage, and ion homeostasis (Ortiz-Rodriguez and Arevalo, 2020). In addition, astrocytes can induce inflammatory or anti-inflammatory processes in microglia, release cytokines, and produce ROS, modulating neuronal redox status and survival (Vicente-Gutierrez et al., 2019; Lee et al., 2021). Noteworthy, under the lipotoxic concentration of PA, human astrocytes present mitochondrial dysfunction, increased superoxide levels, and apoptosis (Vesga-Jiménez et al., 2022a,b). Therefore, these ncRNA interactions have the potential to regulate the CNS protective or deleterious processes derived from lipid imbalance in astrocytes.

However, it is important to establish that mature and immature astrocytes present different morphology, gene expression, functions, and response to injuries, being immature astrocytes less prone to scar formation (Smith et al., 1986; Li et al., 2019). Therefore, as NHA cells are fetal astrocytes, it is possible that the transcriptomic pattern obtained and analyzed in this study does not represent the exact response of astrocytes in an adult human brain. Nevertheless, astrocytic maturation can be induced during cell culture through multiple passages, using growth factors and supplements, or even via astrocyte-to-astrocyte contact (Li et al., 2019). In our case, we verified the expression of developmental and functional genes, including *ALDH18A1*, *SOX9*, *GFAP*, *GJB2*, and *SLC1A3* (Lattke et al., 2021) in PA-treated and VH astrocytes, confirming a certain level of maturation (Supplementary Table 6). Furthermore, the *in vitro* approach may not reflect what happens in a human body, where the interaction with other cells and systems would affect the outcome of the studied lipotoxic conditions. However, the ethical implications of working with patients or obtaining astrocyte cells from them justify the use of human fetal astrocytes to identify the ceRNA networks controlling the astrocytic response to lipotoxicity.

Integrative approaches are currently used to explore the controlling ncRNA network behind cellular processes and NDs. For example, a miRNA–mRNA regulatory network was identified in ROS-induced astrocytic DNA damage, obtaining 231 downregulated and 2 upregulated miRNAs (Nwokwu et al., 2022). The functional enrichment analysis of this miRNA–mRNA network showed an association with signaling, cell cycle, and DNA damage and repair and emphasized the importance of miR-1248, whose inhibition restores the human base-excision repair enzyme hOGG1 (Nwokwu et al., 2022). Additionally, computational methods have also been used to integrate the ncRNA expression during the neuron–astrocyte crosstalk, helping to understand the interaction mechanisms of neuropathological viruses (Selinger et al., 2022).

An improvement this study brings to the neuroscience field related to the ncRNA study is the use of specific lncRNA transcripts, which can present different molecular functions by changing their scaffold properties (Khan et al., 2021). Unfortunately, most of the studies about the importance of lncRNAs in ND cellular mechanisms do not consider which lncRNA transcript was analyzed, and the lack of this crucial information can lead us to biased conclusions that could justify some contradictory studies where the same lncRNA is related to neuronal protection and injury. Therefore, this additional grade of complexity is another reason for the use of computational methods. In this study, we discriminated among lncRNA transcripts, and interestingly, they presented a contrary expression and different miRNA interactions, as in the case of MEG3 ENST00000398461 and ENST00000648820 transcripts.

In this study, five lncRNA transcripts were involved in the 22 obtained axes: MEG3 (ENST00000398461), MEG3 (ENST00000648820), MIR22HG (ENST00000334146), AC092687.3 (ENST00000606907), and SDCBP2-AS1 (ENST00000446423). These axes specifically represent instances where the lncRNAs functioned as miRNA sponges. Consequently, we considered only those mRNAs whose expression levels aligned with the corresponding lncRNA, exhibiting either concurrent upregulation or downregulation. To ensure the coherence of the selected axes within the studied model, we applied a filter to exclude mRNAs and their associated lncRNAs that exhibited opposite regulation. The rationale behind this filtering process stems from the understanding that gene expression is influenced by a multitude of epigenetic and posttranscriptional factors (Corbett, 2018; Cavalli and Heard, 2019). It is highly probable that these alternative mechanisms play a role in modulating the expression of these specific mRNAs, even in scenarios where the miRNAs responsible for their degradation are present or absent.

Interestingly, previous studies have correlated the MEG3 upregulation with improved cognitive impairment and protection against apoptosis in an AD rat model, enhancing spatial learning and memory capability (Yi et al., 2019). However, the role of MEG3 in neuronal protection seems to be condition/transcript-dependent. For example, in the middle cerebral artery occlusion (MCAO) mice model, MEG3 knockdown confers protection in ischemic neuronal death, improving neurological functions through the MEG3/miR-21/PDCD4 and MEG3/miR-424-5p/Sema3A axes (Yan et al., 2017; Xiang et al., 2020). Moreover, MEG3 also regulates miR-378 suppression activity over GRB2, inducing neuronal death, autophagy, and functional impairment (Luo et al., 2020). Additionally, in the MCAO rat model, MEG3 acted as a molecular sponge of miR-485, upregulating AIM2, pyroptosis, and inflammation (Liang et al., 2020). This MEG3 transcript dependency is apparently the case of ENST00000398461 and ENST00000648820, which have opposed expression and regulate different pathways, and further studies will be needed to understand their joint role in astrocyte lipotoxicity. Having 3,452 and 1,113 nucleotides, respectively, these transcripts only coincide in one 34-nucleotide-length exon (Supplementary Figure 1), and this significant contrast in their sequences is probably translated into different biological roles. The fact that neuroscience studies do not consider which MEG3 transcript was analyzed could change the meaning of every conclusion obtained about this lncRNA.

According to our Panther analysis, FGF and epidermal growth factor (EGF) signaling pathways were the most enriched into the “pathways” ontology source for MEG3 (ENST00000398461). These factors are fundamental for nervous system development, maintenance, and repair, regulating differentiation and improving the survival rate of dopaminergic neurons (Romano and Bucci, 2020; Liu Y. et al., 2021). The correlation between these pathways and astrocytic reactivity and CNS injury has been reported, where FGF is required in astrocytes to remain non-reactive (Kang et al., 2014) and EGF for becoming reactive (Liu and Neufeld, 2007). Therefore, the differential regulations of the FGF and EGF pathways in astrocytes are mechanisms leading to CNS protection or damage. Regarding the mirPath analysis, fatty acid metabolism and biosynthesis were highly enriched by the miRNAs controlled by MEG3 (ENST00000398461), while steroid biosynthesis is probably regulated by MEG3 (ENST00000648820) through its interaction with hsa-miR-106a-5p. Fatty acid metabolism in astrocytes has demonstrated a crucial role in AD, where PA induces ceramide *de-novo* synthesis, increasing Aβ production and tau hyperphosphorylation (Patil et al., 2007). On the other hand, steroids have been related to neuroprotection and are considered suitable candidates to improve AD pathology, including neurogenesis, neuroinflammation, mitochondrial impairment, and memory loss (Akwa, 2020). Thus, the two MEG3 transcripts obtained here seem to have an opposite effect on astrocytes, and while ENST00000398461 could be related to harmful mechanisms, ENST00000648820 is probably neuroprotective.

MIR22HG has been involved in the regulation of FoxO and TGF-β signaling pathways (Xu et al., 2020; Chen et al., 2022). Noteworthy, FoxO is related to protection against age-progressive axonal degeneration, and this transcription factor suppression increases white matter astrogliosis and microgliosis (Hwang et al., 2018). Nevertheless, FoxO phosphorylation is observed in AD pathogenesis, promoting ROS production triggered by Aβ (Smith et al., 2005). On the other hand, the TGF-β signaling pathway is underregulated during AD, where SMAD2, SMAD3, and SMAD4, signal transducers in the pathway, have been decreased in the temporal cortex of patients with this disease (Ueberham et al., 2012). Furthermore, it has also been proposed that this pathway alteration in neurons contributes to the accumulation of Aβ, the activation of microglia, and, thus, the development of neurodegeneration (Tichauer and von Bernhardi, 2012). Additionally, MIR22HG showed significative enrichment in prion diseases according to our mirPath analysis. A current hypothesis regarding the propagation of Aβ, tau, and α-synuclein misfolding proposes that these molecules share biophysical and biochemical characteristics with prions (Frost and Diamond, 2009). Interestingly, multiple *in vitro* and *in vivo* studies have described the mechanism of abnormal α-synuclein aggregation, reinforcing this hypothesis in PD (Ma et al., 2019).

AC092687.3 is a very unexplored lncRNA, and its presence and importance have been identified in laryngeal squamous cell carcinoma immunity (Qian et al., 2022) and dilated cardiomyopathy (Zhang H. et al., 2020). Nevertheless, there is no direct relation with NDs currently documented. According to the KEEG analysis in mirPath, the miRNAs probably controlled by this lncRNA transcript highly enriched the fatty acid biosynthesis pathway. Interestingly, an unbalanced diet with high saturated fatty acids can increase the biosynthesis of these molecules, aggravating lipotoxicity conditions (Carta et al., 2017). Therefore, AC092687.3 could be fundamental for astrocytes to control lipotoxic processes in NDs.

Our Panther analysis of the SDCBP2-AS1-related genes revealed their strong association with apoptotic processes, where each ontology source presented terms relative to apoptosis. In ovarian cancer, this lncRNA showed protection against apoptosis, and its suppression impaired cell viability (Liu X. et al., 2021). Interestingly, after further analysis of the transcriptomic expression profile in the apoptosis and p53 KEGG pathways, we observed upregulation in branches of these pathways that lead to cell survival. In contrast, the cell death branches are enriched by the downregulated genes (Supplementary Figure 2). Therefore, SDCBP2-AS1 shows strong potential for apoptosis protection, which is highly interesting under lipotoxic conditions. The SDCBP2-AS1 upregulation could be an attempt to counteract the apoptotic processes induced by PA in astrocytes (Wang et al., 2012; Wong et al., 2014). However, the SDCBP2-AS1 (ENST00000446423)-specific transcript role in astrocytic apoptosis needs further study. In addition, the ECM–receptor interaction term was highly enriched in the mirPath analysis. ECM molecules play an important role in neurodegeneration, modulating neurogenesis, survival, and plasticity. Moreover, ECM can affect the synapse morphology and function and induce or maintain long-term potentiation (Bonneh-Barkay and Wiley, 2009). Noteworthy, on the other hand, integrins, as ECM receptors, have shown a relation with neuroplasticity, modulating ion channels, and reorganization of cytoskeletal filaments (Wu and Reddy, 2012). In addition, it is important to note that the high quantity of cancer terms we found in the mirPath enrichment analysis of these and the other interacting miRNAs could be related to the huge amount of data from cancer studies this miRNA field has.

Respecting SERTAD4-AS1 (ENST00000437764), the miRNAs interacting with this lncRNA enriched the fatty acid biosynthesis and other metabolism-related pathways, which is expected as a lipotoxic response. The regulation of these terms has been observed in the proteomic analysis of NHA cells treated with PA (Vesga-Jiménez et al., 2022a,b), and its relevance has been corroborated in the astrocytic genome-scale reconstructions we developed (Martín-Jiménez et al., 2017; Angarita-Rodríguez et al., 2022). The effect of PA on astrocytes triggering mitochondrial and endoplasmic reticulum stress could be related to the neurodegenerative consequences this fatty acid has (Vesga-Jiménez et al., 2022a,b), and therefore, the role of SERTAD4-AS1 (ENST00000437764) as the fatty acid-related lncRNA needs to be determined.

Interestingly, our enrichment analysis contained shared terms among upregulated and downregulated lncRNA transcripts. For example, the gonadotropin-releasing hormone receptor pathway was overrepresented in both conditions. The decline in gonadal reproductive hormones with age is functionally linked to neurodegeneration (Wang et al., 2010). Similarly, the Ras signaling pathway is also enriched in upregulated and downregulated transcripts. The Ras superfamily regulates crucial processes, including apoptosis, cell survival, and long-term potentiation (Sastre et al., 2020). Interestingly, according to our transcriptomic data, among the DE genes in the Ras signaling pathway, 50% were upregulated and 50% were downregulated (Supplementary Figure 3). Moreover, immune-related pathways were overrepresented in both situations as well, and inflammatory pathways mainly present upregulated genes (Supplementary Figure 4). Altered immune function is associated with reactive gliosis, glial proliferation, cytokine and chemokine release, ROS production, and decreased aggregate clearance, causing synaptic loss, neuronal death, intracellular and extracellular aggregates, and lipid accumulation (Hammond et al., 2019). These shared pathways demonstrate how intricately the ceRNA networks regulate the astrocytic response under lipotoxicity, where different lncRNA transcripts can control the same pathways positively or negatively at multiple points. On the other hand, it is interesting that the downregulated lncRNAs enriched the extracellular exosome GO term. One study has already associated reduced exosome release in astrocytes with elevated levels of cellular lipids (Abdullah et al., 2021). Furthermore, astrocyte-derived exosomes are one of the most important communication pathways between astrocytes and surrounding cells, influencing synaptic plasticity, neurogenesis, neuronal protection, and stress response (Xin et al., 2017; Saeedi et al., 2019). Therefore, this sub-represented GO term could be related to a negative lipidic regulation hampering cellular crosstalk and leading to neurodegenerative processes.

Regarding the five axes supported by the miRNA expression in databases with ND information and ND studies, MEG3 (ENST00000398461)/hsa-let-7d-5p/*ATF6B* and MEG3 (ENST00000398461)/hsa-let-7 g-5p/*ATF6B* were supported by miTED, thus showing the upregulation of these miRNAs in PD. In addition, hsa-let-7d-5p was upregulated in the GSE46131 late AD study. Therefore, MEG3 (ENST00000398461)/hsa-let-7d-5p/*ATF6B* is potentially related to different NDs. *ATF6B* is a cAMP-dependent transcription factor activated during endoplasmic reticulum stress, activating unfolded protein response (Haze et al., 2001; Correll et al., 2019). Furthermore, ATF6β is a transmembrane protein released under stress situations, being translocated to the nucleus, forming homodimers or heterodimers with ATF6α (Haze et al., 2001). Interestingly, ATF6β in the hippocampus regulates the calreticulin (CRT) expression, and the ATF6β-CRT axis promotes neuronal survival during excitotoxicity and endoplasmic reticulum stress (Nguyen et al., 2021). Thus, the downregulation of *ATF6B* as the consequence of the MEG3 (ENST00000398461)/hsa-let-7d-5p, hsa-let-7 g-5p/*ATF6B* axes would imply a failure in this protective mechanism. Reinforcing this idea, the *ATF6* overexpression reduced the expression of the amyloid precursor protein, the level of Aβ_1-42_, and the BACE1 activity (Du et al., 2020).

On the other hand, although the AC092687.3 (ENST0000606907)/hsa-let-7e-5p/[*SREBF2*, *FNIP1*, *PMAIP1*] axes were not supported by the miTED, which does not contain AD data, the AD studies GSE46579 and GSE48552 corroborate the reduction of hsa-let-7e-5p in AD. Thus, AC092687.3 (ENST0000606907)/hsa-let-7e-5p/[*SREBF2*, *FNIP1*, *PMAIP1*] could be related to AD development or pathology. PMAIP1, regulated by the AC092687.3/hsa-let-7e-5p/*PMAIP1*, is a proapoptotic protein that interacts and neutralizes the antiapoptotic MCL1 and BCL2A1 proteins (Oda et al., 2000), regulating autophagic cell death (Elgendy et al., 2011). *PMAIP1* can be induced by multiple stress signals in a p53-dependent or independent manner, depending on the cell type and stress signal (Roufayel, 2016; Sharma et al., 2018; Janus et al., 2020; Roufayel et al., 2022). Therefore, the activation of the AC092687.3/hsa-let-7e-5p/*PMAIP1* axis and, consequently, the *PMAIP1* overexpression would probably induce astrocyte apoptosis. Additionally, FNIP1, as part of the FLCN/FNIP1/FNIP2 complex, acts as an inhibitory regulator of AMPK (Baba et al., 2006; Siggs et al., 2016; Reyes et al., 2021). AMPK is a key regulator of cellular energy balance that helps align the supply of nutrients with the energy needs of cells in mammals, maintaining energy homeostasis. Under conditions of energy stress, AMPK is activated, suppressing energy-intensive biosynthetic pathways such as fatty acid biosynthesis (Viollet et al., 2010). However, this mechanism can fail. For example, PA-induced lipotoxicity can inhibit AMPK, resulting in an increase in malonyl-CoA levels, suppression of CPT-1, and accumulation of fatty acids, exacerbating the initial condition (Drosatos et al., 2013; Fadó et al., 2021). Hence, the *FNIP1* upregulation may lead to a failure of the AMPK mechanism and subsequent metabolic disturbances in astrocytes, exacerbating lipotoxicity and generating ROS accumulation, autophagy inhibition, and apoptosis induction (Unger et al., 2010). Furthermore, *SREBF2* was also controlled by the AC092687.3 (ENST00000606907)/hsa-let-7e-5p axis. This gene codifies a transcription factor recognizing sterol regulatory element 1 (SRE-1) and regulating cholesterol homeostasis (Hua et al., 1993). In addition, this transcription factor is involved in aberrant tau phosphorylation and Aβ production mediated by astrocytic cholesterol production (Wang et al., 2021b; Glasauer et al., 2022). Thus, the regulation of *SREBF2* gene expression would be crucial in CNS cholesterol dyshomeostasis, leading to neurodegeneration.

Finally, only hsa-miR-101-3p in the SDCBP2-AS1 (ENST00000446423)-related axes was supported by at least two studies (GSE46579 and GSE48552), both related to AD. Therefore, the SDCBP2-AS1 (ENST00000446423)/hsa-miR-101-3p/*MAPK6* axis seems to be important for AD development and pathology. MAPK6 participates in the MAPK signaling pathway, which is related to cellular proliferation and differentiation, inflammation, and apoptosis (Wei and Liu, 2002). In penicillin-induced astrocytes, an *in vitro* seizure model MAPK6 demonstrated importance in apoptosis and inflammation since its overexpression reduces cell viability and upregulates TNF-α/IL-1β expression (Pang et al., 2022). Therefore, *MAPK6* positive regulation as a consequence of the SDCBP2-AS1 (ENST00000446423)/hsa-miR-101-3p/*MAPK6* axis would be deleterious in AD.

In conclusion, the present study has presented an extensive ceRNA network that would control how astrocytes react to high PA concentrations, which can be very useful in understanding the mechanisms protecting or injuring the CNS under lipotoxic conditions. This ceRNA network is part of the intricated epigenetic regulation at the cellular level, which could influence the pathology of different NDs. Interestingly, the MEG3 (ENST00000398461)/hsa-let-7d-5p/*ATF6B*, AC092687.3 (ENST0000606907)/hsa-let-7e-5p/*SREBF2*, AC092687.3 (ENST0000606907)/hsa-let-7e-5p/*FNIP1*, AC092687.3 (ENST0000606907)/hsa-let-7e-5p/*PMAIP1*, and SDCBP2-AS1 (ENST00000446423)/hsa-miR-101-3p/*MAPK6* axes showed probable importance in AD and PD and were corroborated with multiple ND studies published in GEO and miTED databases. Due to the *in vitro* character of this study and its high *in silico* component, further functional studies are required to determine the role of these molecules in PA-induced astrocytic stress and the resulting CNS injuries. If their interesting features are conserved at the *in vivo* level, these axes could be studied as targets for new pharmacologic treatments or as possible diagnosis molecules, improving the quality of life of millions around the world.

## Data availability statement

The original contributions presented in the study are included in the article/Supplementary material, further inquiries can be directed to the corresponding author.

## Author contributions

NG-J, AA-P, AP, and JG: conceptualization. NG-J, AA-P, AR-C, YG-G, AP, and JG: methodology. NG-J, AA-P, ML, VG, HE, LG, JJ, and AR-C: formal analysis. AA-P, AP, and JG: resources. NG-J, ML, VG, HE, LG, JJ, and AR-C: writing—original draft. NG-J, AA-P, YG-G, AP, and JG: writing—reviewing and editing. NG-J: visualization. AA-P, AP, and JG: supervision and funding acquisition. All authors contributed to the article and approved the submitted version.
