# Supplementary material for: Competing endogenous RNAs in human astrocytes: crosstalk and interacting networks in response to lipotoxicity
Source: Front Neurosci. 2023 Nov 13;17:1195840. doi: 10.3389/fnins.2023.1195840 (PMC10679742; doi:10.3389/fnins.2023.1195840)
Supplement: Supplementary file 7 [file Data_Sheet_1.pdf]

Supplementary Material

Competing endogenous RNAs in human astrocyte: crosstalk and interacting networks in response to lipotoxicity

Natalia Gil-Jaramillo[1], Andrés Felipe Aristizábal-Pachón[1], María Alejandra Luque Aleman[1], Valentina González Gómez[1], Hans Deyvy Escobar Hurtado[1], Laura Camila Girón Pinto[1], Juan Sebastian Jaime Camacho[1], Alexis Felipe Rojas-Cruz[1], Yeimy González-Giraldo[1], Andrés Pinzón[2], and Janneth González[1, *]

[1]Departamento de Nutrición y Bioquímica, Facultad de Ciencias, Pontificia Universidad Javeriana, Bogotá, Colombia

[2]Laboratorio de Bioinformática y Biología de Sistemas, Universidad Nacional de Colombia, Bogotá, Colombia

*** Correspondence:**
Janneth González
janneth.gonzalez@javeriana.edu.co

1 Supplementary Figures and Tables

1.1 Supplementary Figures

A

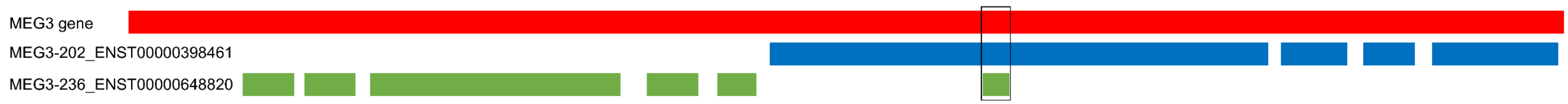

B

```
MEG3     55201 CCCTCTCTTTGTCCCTCCCCAGTTCCTGACCTGGCCATCCCGGGGTGCCCTTGACCAGCCCCGTGTCTCCTCAGGGTGTCCCAGCACCAGCCTGGCACAGAGTGGGGCTCAGTTAGAGTA 55320
MEG3-202 55201 CCCTCTCTTTGTCCCTCCCCAGTTCCTGACCTGGCCATCCCGGGGTGCCCTTGACCAGCCCCGTGTCTCCTCAGGGTGTCCCAGCACCAGCCTGGCACAGAGTGGGGCTCAGTTAGAGTA 55320
MEG3-236 55201 ---------------------------------------------------------------------------GGTGTCCCAGCACCAGCCTGGCACAGAGTGGG--------------- 55320
```

C

```
>MEG3-202_ENST00000398461_Exon1

CATTGAGGACATCCAAAACTCACTCAAGATCACCAAGTGGTAGGAAAGTACTAACTCCTGGGCATAGCCCTAGGGGAGTGACTACAATGTGAATACTCATGG
AATGCCTAGCCAGGTGAAGAAGTGAATGCATGTTGGCATCCCAGAGGGACCCCCCTTAAGAGGGCATAGTTTGGGGTTCAGATTTGACTCCAGCATACTGTT
GAAATTGGGCACAGGGGGCTAGTGATTAGTCAGGATCAATCAGTGGAGAGGAGATTAAAACTCACATCTGGGAGTCCGGAATCAGAACTTGTAGTTCTTTTT
TTTGAAATGGAGTCTCGCTCTGTCGCCCAGGCTGGAGTGCAGTGGCGTGATCTCGGCTCACTGCAACCTCTGCCTTCCGGGTTCAAGCAATTCTCCCACCTC
AGCCTCCTGAGTAGCTGGGATTACAGGCGCCCACCACCACAGCCGGCTACTTTTTGTATTTTTAGGAGAGACGGGGTTTCACCATGTTGGTCAGGCTGGTCT
TGAACTCCTGACCTCATGATCCACCTGCCTTAGCCTCCCAAAGTGCTGGGATTACAGGCGTGAGCCACCATGCCCAGCCCTATAGTAGTTCTTCTTTTGCCC
CTTAATATCTTCACCCACATGTCCTGTACCCTGCCTGAACCCTCCTCCTCTTTTTGTTCTGATCTTTGAGCTCCCTAGAGCCCATAATTCTTTAGAGCAGGT
ATGTCCCGAGTCTGAAACATGCCCTTATTTGTCCCAAGCTCTGGACATTTCTCACCCCAAGGCGGATCAATCATGATTAAATCACTCCAATTAAACTTTAGG
CTCCAGTCAGACCTTCAGCCAAATGGAAAAAAAAACTAGGGGATAAGGGAGGTAGTTGGAGCAAGAAAATGTTATTAGTTGAAACCTTACGGGACCTTCCTC
CCTTAGTGAGTCTGTTGGCTAAAGGTTCTCTGGCTTCGTGAATTAGAATCGGATACTGTTTCCAAGTTAGCAAAACCAACTCTACCCCAGCACCCCACGAGG
AAGAATGTGGAAGGATCTCCCATTGGCCGGTTGGGGCAAAAGCCTGAGGCAATCTTTCATCCCCTTTTGCCAAGGCGAGACTTTCCCAGTGACGGTGATGTA
GTTGGCCACTCTGACTATGGGTGGACTCGGGTGTAGACCTCTGAAGCTGAGATCACACGAAAACCTGGCCTCCCCGCCATGTAGCTGTTGGAGAGTAGAAAA
ATAGAGCACGCCTGATGTTTCTAAATGAGAAGACTTTCAATAGTAATGAAGAATCCATGGCACTCTCCTCACCCTCAAACACATGGCAGTCATTCACATACA
GGCCCCAAAGCCACTGTTAGTGCTGCAGTAGCTCCTGTGGACATTGGAAAGCCCGGAGAGGGCGTGGAAGAAATCAGCTGGCCCCCGGCAGGTTCTCTGGGG
TTTTGTGCCCAAGGCTCCTGGAGCCCTAAAAACTTTCAAAAGTTAACTCCCCACGTCCCCATCCTGCTTGGGTTTCTGGACTTTTCTGAGGCACCGGCAGAG
GGGTCTCGTTGCTCCCTTGAGTGTAGGGGCAGCCCTTTAACCTGGCTCCTTGAGTCCCTGCTTTTTCTGCTTCTGTTGCCTTCTTCCTCGTCTTCCTCTCTC
TCAATATCTCCCTCTCTTTGTCCCTCCCCAGTTCCTGACCTGGCCATCCCGGGGTGCCCTTGACCAGCCCCGTGTCTCCTCAGGGTGTCCCAGCACCAGCCT
GGCACAGAGTGGGGCTCAGTTAGAGTATGTGGGATGTTGGTTTCGCCAGGTGAGTGAATGAAAGGACTCGACCACCACAGCTGAGCCACTAGCTGGGCCATG
CGAAGAGTTCTAGGTGCAAAGGCTGGAGGGTGGAATTCATTTTTGAGAGGTGTGTGAGCAGCTTCCGACCCCTGCCCCATTTGAACGGGGGCCTTGCTGGTC
GCGTCCCTGCATTCACCCGCGCGGCCATCCCGTCATCCAACAGTTGATCCTAACTGAGCACGCCCACGGCCCTGGTCTGGCCTGGGCACCGGCCACCGTAGC
CCATCCCTTGATGGCCTCTGTGTCCCCAGGAGGGCGGGCCGGGGGGTTGCCCAGGGGCTGGAGCAGTGGACTGTGGCTCCATAGAGGTAGGCCGGAGGGTGT
```

GAGGGCAGATTCAAGCTATCCCCAGGGCTCTGCTCTGGTCGGAGCCAGCCCCTTCTCCCTCTCTGCCTTCCCCGCCCCATTCCTGATGCTGAACTGTTCTGG
ACCCCTGGCCCTGAGTCTCTCAGGACCAAAGTGGGCACGGGAACAGCTGTAGTGTGTGCCCCCCCGGGCTTTGGCCACAGGTCTCCCTCTCGAGGTGTGGTT
GTGACTGCGACCCTTCCCTTGCCGTGATGCCTTCCTCCCCCGGGGCTTGGTCCAGCTCCTTCACTCTCTAGCAGCTGCTGGGGCCCACCTCCCATGCCGAGG
ACCAGCAGGGGAAACCTCCAGGGAGCATCTGCAGGCTCTGCTTCTGCCCGGCTGCTGGCTTGCTCTCCCTGGTGGCTCTCCAGCGGCCAGCTTCCTCACCCA
CCCGGCACTCCGCTTTGCTCTGTCTCCTGAGGTGGGCCTGACCAACCTCCCCTTCTCTGCCTCAGTCCCTGGGCTCCAGGGCTCAGCTCCACAGCCCTCTGC
CTAGCAGGCTGGTTCTCCCTGCCAAGCCCATACCTGTGGTCACCTGGCCCTCCTGTGGTCTGAGTACCACTCCCCTGCCCCAGGAGCCACTCCCACTCCAGC
TGCCTGTTTCCAGCAGGTTCCCAGTGCCCCCGACAAGCCCCTGCTGGTGTCTCCATCTCCTGCCAAGCATCCTCCAGTGCCTCCTCCTGTGGGCCTGGCCTC
AGGGCTATGGACAGACTCCTGTCCCATCCCAGAGACCCCTCGTGATCGTGCCCTG

>MEG3-202_ENST00000398461_Exon2

GCACGTGGGCCGTGGCCCGGCTGGGTCGGCTGAAGAACTGCGGATGGAAGCTGCGGAAGAGGCCCTGATGGGGCCCACCATCCCGGACCCAAGTCTTCTTCC
TGGCGGGCCTCTCGTCTCCTTCCTGGTTTGGG

>MEG3-202_ENST00000398461_Exon3

CGGAAGCCATCACCTGGATGCCTACGTGGGAAGGGACCTCGAATGTGGGACCCCAGCCCCTCTCCAGCTCGAAATC

>MEG3-202_ENST00000398461_Exon4

CCTCCACAGCCACGGGGACACCCTGCACCTATTCCCACGGGACAGGCTGGACCCAGAGACTCTGGACCCGGGGCCTCCCCTTGAGTAGAGACCCGCCCTCTG
ACTGATGGACGCCGCTGACCTGGGGTCAGACCCGTGGGCTGGACCCCTGCCCACCCCGCAGGAACCCTGAGGCCTAGGGGAGCTGTTGAGCCTTCAGTGTCT
GCATGTGGGAAGTGGGCTCCTTCACCTACCTCACAGGGCTGTTGTGAGGGGCGCTGTGATGCGGTTCCAAAGCACAGGGCTTGGCGCACCCCACTGTGCTCT
CAATAAATGTGTTTCCTGTCTTAAC

D

>MEG3-236_ENST00000648820_Exon1

GGCGGAGAGCAGAGAGGGAGCGCGCCTTGGCTCGCTGGCCTTGGCGGCGGCTCCTCAGGAGAGCTGGGGCGCCCACGAGAGGATCCCTCACCCG

>MEG3-236_ENST00000648820_Exon2

GGTCTCTCCTCAGGGATGACATCATCCGTCCACCTCCTTGTCTTCAAGGACCACCTCCTCTCCATGCTGAGCTGCTGCCAAGGGGCCTGCTGCCCATCTACA

CCTCACG

>MEG3-236_ENST00000648820_Exon3

AGGGCACTAGGAGCACGGTTTCCTGGATCCCACCAACATACAAAGCAGCCACTCACTGACCCCCAGGACCAGGATGGCAAAGGATGAAGAGGACCGGAACTG
ACCAGCCAGCTGTCCCTCTTACCTAAAGACTTAAACCAATGCCCTAGTGAGGGGGCATTGGGCATTAAGCCCTGACCTTTGCTATGCTCATACTTTGACTCT
ATGAGTACTTTCCTATAAGTCTTTGCTTGTGTTCACCTGCTAGCAAACTGGAGTGTTTCCCTCCCCAAGGGGGTGTCAGTCTTTGTCGACTGACTCTGTCAT
CACCCTTATGATGTCCTGAATGGAAGGATCCCTTTGGGAAATTCTCAGGAGGGGGACCTGGGCCAAGGGCTTGGCCAGCATCCTGCTGGCAACTCCAAGGCC
CTGGGTGGGCTTCTGGAATGAGCATGCTACTGAATCACCAAAGGCACGCCCGACCTCTCTGAAGATCTTCCTATCCTTTTCTGGGGGAATGGGGTCGATGAG
AGCAACCTCCTAGGGTTGTTGTGAGAATTAAATGAGATAAAAGAGGCCTCAGGCAGGATCTGGCATAGAGGAGGTGATCAGCAAATGTTTGTTGAAAAGGTT
TGACAGGTCAGTCCCTTCCCACCCCTCTTGCTTGTCTTACTTGTCTTATTTATTCTCCAACAGCACTCCAGGCAGCCCTTGTCCACGGGCTCTCCTTGCATC
AG

>MEG3-236_ENST00000648820_Exon4

CCAAGCTTCTTGAAAGGCCTGTCTACACTTGCTGTCTTCCTTCCTCACCTCCAATTTCCTCTTCAACCCACTGCTTCCTGACTCGCTCTACTCCGTGGAAGC
ACGCTCACAAAG

>MEG3-236_ENST00000648820_Exon5

GGCTAATCTCGGGCCTTGTCGAAGGAAGAGGCTGCAGACGTTAATGAG

>MEG3-236_ENST00000648820_Exon6

GGTGTCCCAGCACCAGCCTGGCACAGAGTGGG

Supplementary Figure 1. MEG3 ENST00000398461 and ENST00000648820 transcript comparison. DE MEG3 transcripts were aligned to understand the biological meaning of their opposed expression level and the probability of having different roles during astrocytic lipotoxicity. (A) Graphic representation of MEG3 gene sequence alignment against ENST00000398461 (also known as MEG3-202, in blue) and ENST00000398461 (or MEG3-236, in green) transcripts. (B) Zoom into the overlapping region. (C) MEG3 ENST00000398461 transcript exons. (D) MEG3 ENST00000648820 transcript exons. The overlapping region is shown in a black square.

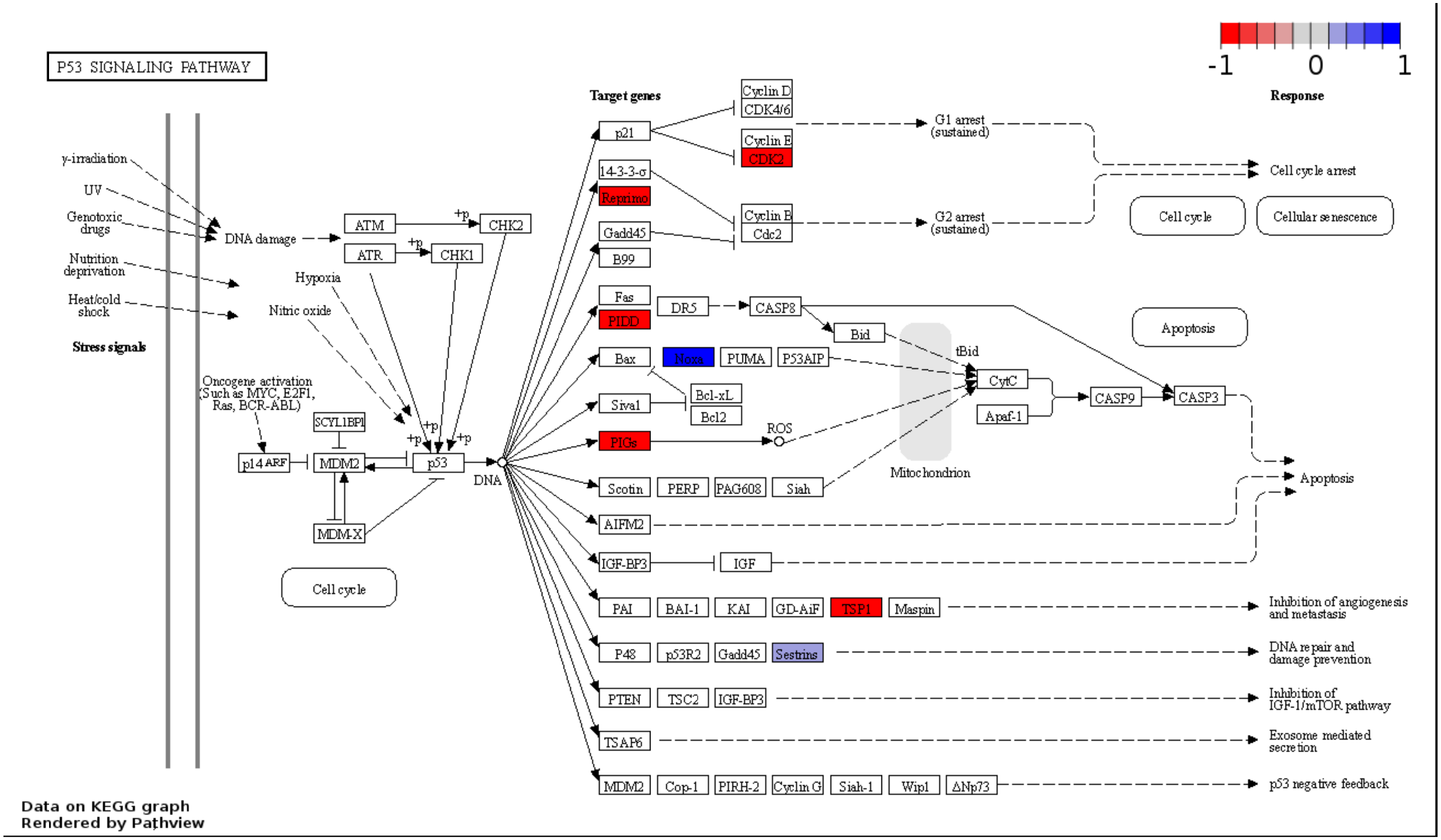

P53 SIGNALING PATHWAY
Response
-1
0
1
Target genes
Stress signals
γ-irradiation
UV
Genotoxic drugs
Nutrition deprivation
Heat/cold shock
DNA damage
ATM
ATR
CHK2
CHK1
+p
Hypoxia
Nitric oxide
Oncogene activation (Such as MYC, E2F1, Ras, BCR-ABL)
SCYL1BP1
p14ARF
MDM2
p53
MDM-X
DNA
Cell cycle
p21
14-3-3-σ
Reprimo
Gadd45
B99
Cyclin D
CDK4/6
Cyclin E
CDK2
Cyclin B
Cdc2
G1 arrest (sustained)
G2 arrest (sustained)
Cell cycle arrest
Cell cycle
Cellular senescence
Fas
PIDD
DR5
CASP8
Bid
tBid
Bax
Noxa
PUMA
P53AIP
Sival
Bcl-xL
Bcl2
PIGs
ROS
Mitochondrion
CytC
Apaf-1
CASP9
CASP3
Apoptosis
Scotin
PERP
PAG608
Siah
AIFM2
IGF-BP3
IGF
PAI
BAI-1
KAI
GD-AiF
TSP1
Maspin
Inhibition of angiogenesis and metastasis
P48
p53R2
Gadd45
Sestrins
DNA repair and damage prevention
PTEN
TSC2
IGF-BP3
Inhibition of IGF-1/mTOR pathway
TSAP6
Exosome mediated secretion
MDM2
Cop-1
PIRH-2
Cyclin G
Siah-1
Wip1
ΔNp73
p53 negative feedback
Data on KEGG graph
Rendered by Pathview

Supplementary Figure 2. p53 signaling pathway enrichment. DE genes in the transcriptome are displayed in the pathway according to their Log_2 Fold change, with dark blue for higher values and dark red for lower values.

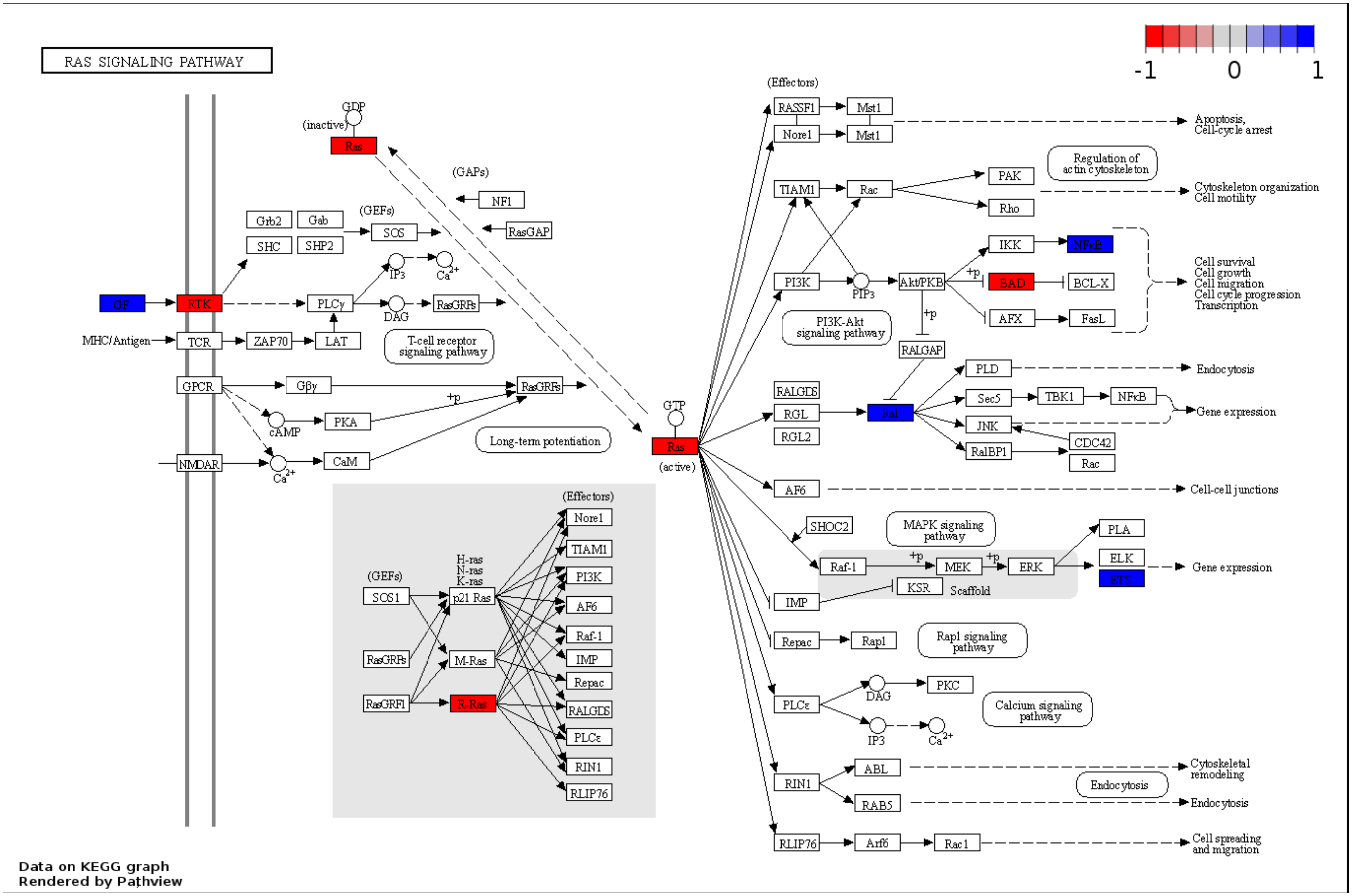
RAS SIGNALING PATHWAY
-1
0
1
GF
RTK
MHC/Antigen
TCR
GPCR
NMDAR
Grb2
Gab
SHC
SHP2
(GEFs)
SOS
PLCγ
ZAP70
LAT
IP3
DAG
RasGRPs
T-cell receptor signaling pathway
Gβγ
cAMP
PKA
CaM
RasGRFs
+p
Long-term potentiation
GDP
(inactive)
Ras
(GAPs)
NF1
RasGAP
GTP
(active)
(Effectors)
H-ras
N-ras
K-ras
p21 Ras
M-Ras
R-Ras
SOS1
RasGRFs
RasGRF1
Nore1
TIAM1
PI3K
AF6
Raf-1
IMP
Repac
RALGDS
PLCε
RIN1
RLIP76
RASSF1
Mst1
Rac
PAK
Rho
Regulation of actin cytoskeleton
Apoptosis, Cell-cycle arrest
Cytoskeleton organization Cell motility
PIP3
Akt/PKB
IKK
NFκB
BAD
BCL-X
AFX
FasL
PI3K-Akt signaling pathway
RALGAP
Cell survival
Cell growth
Cell migration
Cell cycle progression
Transcription
RGL
RGL2
Ral
PLD
Sec5
TBK1
JNK
RalBP1
CDC42
Endocytosis
Gene expression
Cell-cell junctions
SHOC2
MAPK signaling pathway
MEK
ERK
KSR
Scaffold
PLA
ELK
ETS
Rap1
Rap1 signaling pathway
PKC
Ca2+
Calcium signaling pathway
ABL
RAB5
Cytoskeletal remodeling
Arf6
Rac1
Cell spreading and migration
Data on KEGG graph
Rendered by Pathview

Supplementary Figure 3. Ras signaling pathway enrichment. DE genes in the transcriptome are displayed in the pathway according to their Log_2 Fold change, with dark blue for higher values and dark red for lower values.

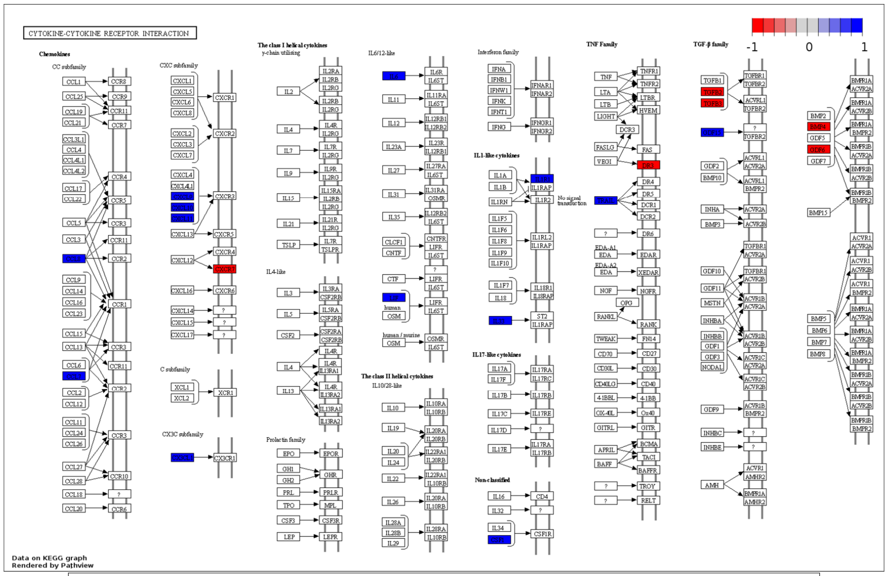
CYTOKINE-CYTOKINE RECEPTOR INTERACTION
-1
0
1
Data on KEGG graph
Rendered by Pathview

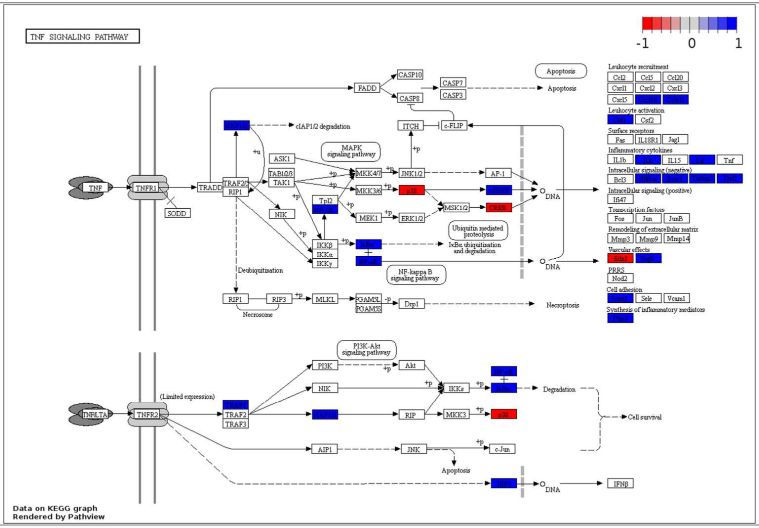
TNF SIGNALING PATHWAY
-1
0
1
Data on KEGG graph
Rendered by Pathview

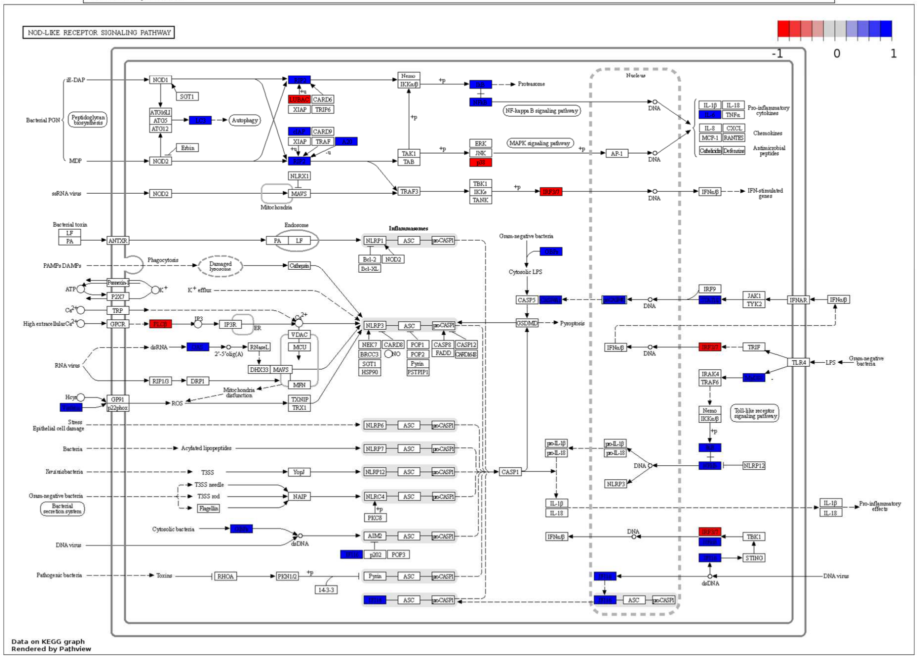
NOD-LIKE RECEPTOR SIGNALING PATHWAY
-1
0
1
Data on KEGG graph
Rendered by Pathview

Supplementary Figure 4. Inflammatory pathways enrichment. DE genes in the transcriptome are displayed in the pathway according to their Log_2 Fold change, with dark blue for higher values and dark red for lower values.

1.2 Supplementary Table Legends

Supplementary Table 1. Primers. Primers used for validation of the selected axis are listed here.

Supplementary Table 2. DE genes in the transcriptome. Sheet tab "PA-DEG_up" displays all the significantly upregulated genes in the transcriptome, while sheet tab "PA-DEG_down" displays all the significantly downregulated genes in the transcriptome. Comparisons were made between PA-treated astrocytes (PA) and vehicle astrocytes (VH). Gene ID, symbol, biotype, Log_2 Fold change, p-value, and FDR-corrected p-value are shown for every gene.

Supplementary Table 3. Complete list of Panther enriched terms. Sheet tabs "MIR22HG (ENST00000334146)", "SDCBP2-AS1 (ENST00000446423)", and "MEG3 (ENST00000398461)" display the whole list of Panther enriched terms from the mRNAs related to MIR22HG (ENST00000334146), SDCBP2-AS1 (ENST00000446423), and MEG3 (ENST00000398461), respectively. Sheet tab "lncRNAs_up" shows the list of Panther enriched terms from the mRNAs related to the positively regulated lncRNAs, while sheet tab "lncRNAs_down" shows the whole list of Panther enriched terms from the mRNAs related to the negatively regulated lncRNAs.

Supplementary Table 4. Complete list of miRPath enriched KEGG pathways. Each sheet tab displays the whole list of enriched terms obtained from the miRNAs that interact with MEG3 (ENST00000396181), MEG3 (ENST00000648820), LINC01503 (ENST00000444125), AC092687.3 (ENST00000606907), SERTAD4-AS1 (ENST00000437764), SDCBP2-AS1 (ENST00000446423), and MIR22HG (ENST00000334146) lncRNAs, respectively. FDR-corrected p-value and the number of related genes and miRNAs are shown for each pathway.

Supplementary Table 5. Biological role of each RNA molecule in the selected axes, according to databases. Relative expression obtained from BioGPS and CNS microRNA profiles is presented as "above," "on," or "under" the median expression of total tissues in the databases.

Supplementary Table 6. Trimmed mean of M-values (TMM) of developmental and functional genes in mature astrocytes. The TMM normalized expression of *ALDH18A1*, *SOX9*, *GFAP*, *GJB2*, and *SLC1A3* genes is displayed for the replicates of AstroPA (PA-treated astrocytes) and AstroVH (VH astrocytes).
